# From Fundamentals to Innovation in Alzheimer’s Disease: Molecular Findings and Revolutionary Therapies

**DOI:** 10.3390/ijms252212311

**Published:** 2024-11-16

**Authors:** Mădălina Georgeta Sighencea, Ramona Ștefania Popescu, Simona Corina Trifu

**Affiliations:** 1 Doctoral School, “Carol Davila” University of Medicine and Pharmacy Bucharest, 020021 Bucharest, Romania; madalina-georgeta.iliescu@drd.umfcd.ro; 2 Department of Infectious Diseases, “Carol Davila” University of Medicine and Pharmacy Bucharest, 020021 Bucharest, Romania; ramona.popescu1@umfcd.ro; 3 Department of Psychiatry, “Carol Davila” University of Medicine and Pharmacy Bucharest, 020021 Bucharest, Romania

**Keywords:** Alzheimer’s disease, neurodegenerative disorder, molecular mechanisms, genome, microbiota, therapeutic approach

## Abstract

Alzheimer’s disease (AD) is a global health concern and the leading cause of dementia in the elderly. The prevalence of this neurodegenerative condition is projected to increase concomitantly with increased life expectancy, resulting in a significant economic burden. With very few FDA-approved disease-modifying drugs available for AD, there is an urgent need to develop new compounds capable of impeding the progression of the disease. Given the unclear etiopathogenesis of AD, this review emphasizes the underlying mechanisms of this condition. It explores not only well-studied aspects, such as the accumulation of Aβ plaques and neurofibrillary tangles, but also novel areas, including glymphatic and lymphatic pathways, microbiota and the gut–brain axis, serotoninergic and autophagy alterations, vascular dysfunction, the metal hypothesis, the olfactory pathway, and oral health. Furthermore, the potential molecular targets arising from all these mechanisms have been reviewed, along with novel promising approaches such as nanoparticle-based therapy, neural stem cell transplantation, vaccines, and CRISPR-Cas9-mediated genome editing techniques. Taking into account the overlap of these various mechanisms, individual and combination therapies emerge as the future direction in the AD strategy.

## 1. Introduction

Alzheimer’s disease (AD) is the primary cause of dementia (60–80%) and one of the main neurodegenerative disorders in the elderly. Along with the expected increase in the elderly population from 703 million in 2015 to 1.5 billion by 2050, there is also an expected increase in dementia cases from 50 million to 152 million worldwide by 2050 [1,2]. Thus, Alzheimer’s disease constitutes a global health concern, resulting in a considerable economic burden. Therefore, by 2030, global dementia costs are estimated to reach USD 2 trillion, with formal caregiving accommodation causing a rise in direct costs of up to 67.3% of the total financial burden of the disease [2].

Until recently, the primary Food and Drug Administration (FDA)-approved treatment for Alzheimer’s disease focused only on symptoms and relied on acetylcholinesterase inhibitors such as rivastigmine, donepezil, and galantamine, in addition to memantine, an NMDA antagonist [3]. To date, the FDA has approved three novel anti-Aβ pharmaceuticals that act as monoclonal antibodies: lecanemab, aducanumab, and donanemab, with the latter receiving approval in July 2024 [4,5]. In the meantime, aducanumab has been discontinued, though not due to issues regarding its safety or efficacy [6]. As a result, these compounds represent the first FDA-approved disease-modifying therapy in AD, with many more molecules currently undergoing clinical trials [4]. Although the precise pathophysiological mechanisms of AD remain uncertain, there has been growing interest in unraveling its pathogenesis to facilitate the development of new potential pharmaceutical interventions [7]. However, the main histopathological hallmarks of this disorder are the aberrant phosphorylation of the tau protein along with the deposition of β-amyloid plaques in the brain [7,8]. In addition to these, numerous other classical mechanisms have been described in AD, including the cholinesterase hypothesis, oxidative stress, advanced glycation and lipid peroxidation products, mitochondrial dysfunction, neuroinflammation, insulin resistance, alterations in insulin signaling pathways, and neuronal cell cycle re-entry [9].

Meanwhile, several other mechanisms are currently being explored, including the glymphatic and lymphatic pathways, alterations in the microbiota and the gut–brain axis, serotoninergic impairments, autophagy alterations, vascular dysfunction, olfactory pathway, oral health, and the metal hypothesis, particularly involving calcium and iron [1,9,10,11,12]. The involvement of genes in AD pathogenesis is also widely studied, with recent extensive genomic analyses revealing more than 40 genetic risk factors that have been implicated in various neuropathological processes [13].

**Figure 1:**
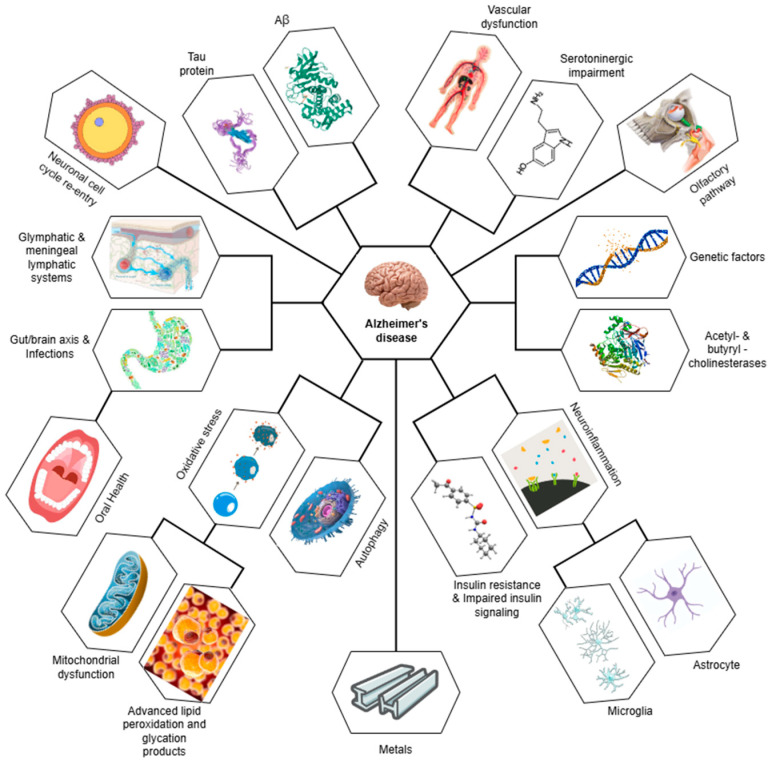
The molecular mechanisms in Alzheimer’s disease pathogenesis.

This review offers a thorough and current overview of the research landscape on the molecular mechanisms underlying AD and their possible therapeutic targets. The identification of relevant studies was primarily based on searches within major academic databases, including Scopus, Nature, ScienceDirect, Wiley, Taylor & Francis, and SpringerLink. Only articles published in English were considered, and duplicate entries were excluded to prevent repetition. Figure 1 below gathers the diversity of mechanisms that have currently been described in AD pathogenesis.

## 2. The Amyloid Pathway

The amyloid-beta (Aβ) has been considered a driver of Alzheimer’s pathological processes. In addition, its accumulation within the brain may precede the emergence of clinical symptoms by up to twenty years. As a result, the “amyloid cascade hypothesis” has become a leading theory of the pathogenesis of AD [14]. According to this theory, the accumulation of amyloid-β within the context of Alzheimer’s disease leads to synaptic malfunction and neurodegeneration [15].

Consequently, beta-amyloid plaque (ABP), predominantly composed of Aβ peptides, is one of the main pathological characteristics of AD [14,15]. It is established that Aβ peptides are generated through the proteolytic cleavage of a type I transmembrane protein named amyloid precursor protein (APP) by several secretases [1]. Two different processing pathways have been identified for the amyloid precursor protein: the non-amyloidogenic pathway and the amyloidogenic pathway [15]. Non-amyloidogenic APP cleavage refers to the initial activity of α-secretase followed by γ-secretase and seems to be neuroprotective [1,8]. On the other hand, the amyloidogenic pathway is characterized by the cleavage of amyloid precursor protein by β-secretase (BACE-1), leading to the liberation of the transmembrane C-terminal fragment (C99), along with soluble APP beta (sAPPβ). Subsequently, the C99 fragment is processed by several subunits of the γ-secretase complex, known as presenilins. As a result, Aβ peptides consisting of 38–43 amino acids are generated [16]. After that, Aβ monomers undergo oligomerization and polymerization, forming amyloid fibrils, which have the ability to aggregate to form Aβ plaques. This process promotes the activation of certain kinases capable of inducing the generation of insoluble neurofibrillary tangles (NFTs) by hyperphosphorylating microtubule-associated proteins [8,9]. 

On the other hand, it is known that in the context of certain pathological changes or aging, β- and γ-secretases may decrease their capacity to degrade Aβ, leading to an accumulation of A40 and A42 peptides [17]. Furthermore, elevated A42/A40 ratios may facilitate the generation of Aβ amyloid fibrils, culminating in neurotoxicity through various mechanisms, such as disrupting cell signaling, affecting synapses, altering membrane permeability, and inducing neuroinflammation, excitotoxicity, mitochondrial dysfunction, and oxidative stress [8,17].

The competition between the amyloidogenic and non-amyloidogenic pathways currently stands as an important therapeutic target in Alzheimer’s disease for mitigating Aβ production [18].

The stability and effects of Aβ plaques in AD differ significantly depending on their type, with diffuse and dense-core plaques affecting the severity of the disease in unique ways. Diffuse plaques, although commonly observed even in elderly individuals with normal cognitive function, are usually less strongly linked to cognitive decline. On the contrary, dense core (or focal) plaques, which are frequently linked to neuroinflammation and tau pathology, have a closer association with cognitive deterioration and the advancement of AD. Research indicates that diffuse plaques may form earlier and develop into dense-core plaques, which are more stable and neurotoxic, playing a role in neurodegenerative processes, inflammatory responses, and more severe cognitive impairment in AD patients [19].

Studies have shown that the progression of AD may be due to the dysregulation between Aβ production and clearance. Therefore, to maintain brain homeostasis, there are various mechanisms that contribute to the elimination of intracellular and extracellular Aβ surplus, including various transport pathways, the autophagy-lysosome system (ALS), several Aβ-degrading enzymes (ADEs), as well as the ubiquitin–proteasome system (UPS). Consequently, augmenting Aβ clearance mechanisms is a promising strategy to mitigate the progression of AD [1].

Targeting Aβ represents a crucial goal in the new therapy for AD. Consequently, there is increasing interest in investigating molecules that could serve as γ-secretase inhibitors, BACE inhibitors, and also as α-secretase modulators. Although γ-secretase inhibitors have shown notable safety concerns, two BACE inhibitors are showing promising results, with elenbecestat currently in phase 2 and umibecestat in phase 3 of clinical trials. Meanwhile, several drugs act as α-secretase modulators by activating the PI3K/Akt pathway and could potentially be effective in treating mild to moderate AD [3]. Another emerging perspective in Alzheimer’s disease treatment involves passive Aβ immunotherapy utilizing monoclonal antibodies. Therefore, in addition to the FDA-approved monoclonal antibodies targeting Aβ, namely aducanumab, lecanemab, and donanemab, numerous other molecules are currently undergoing clinical and preclinical trials, such as crenezumab, gantenerumab, and solanezumab [3,4]. Immunotherapeutic approaches in AD treatment also encompass active Aβ immunotherapy, which involves several compounds acting as Aβ vaccines. These are currently being studied in various phases of research [3].

## 3. Tau Protein

After amyloid plaques, neurofibrillary tangles constitute the second most prevalent histopathological feature of AD. These may be generated through the aggregation of tau protein. It refers to a microtubule-associated protein primarily responsible for dendritic organization, axonal transport, and the preservation of microtubule structural stability. Moreover, its functions have been shown to be influenced by post-translational alterations, such as phosphorylation at numerous sites [8]. Various studies have shown that APP can propagate tau accumulation, implying that increased APP production in brains with Alzheimer’s disease may be associated with tau pathology [20]. Furthermore, recent studies have revealed intriguing insights into potential novel mechanisms for the transmission of abnormal tau proteins between cells, including prion-like and exosome-mediated pathways. These findings may contribute to a better understanding of the propagation of tau aggregates within the brain [21,22,23].

In addition, AD has been evidenced to be marked by the hyperphosphorylation of tau protein, particularly at threonine, tyrosine, and serine residues, enhancing the protein’s susceptibility to aggregation. Concurrently, the abnormal phosphorylation of tau disrupts microtubule integrity, leading to diminished synaptic connectivity, altered dendritic architecture, impaired axonal transport, neuronal apoptosis, and ultimately resulting in dementia [8,17,24]. Moreover, tau phosphorylation at specific sites such as Ser202, Thr205, Thr231, Thr217, and Thr181 may serve as cerebrospinal fluid (CSF) biomarkers for the preclinical stage of AD [25,26]. The excessive phosphorylation of tau in brains impacted by AD can result from both conformational changes in tau, making it more susceptible to phosphorylation, and a disproportion between protein kinases and protein phosphatases 1 and 2A (PP1 and PP2A). Consequently, the main kinase enzymes implicated in the phosphorylation of tau consist of glycogen synthase kinase-3 (GSK-3), protein kinase A (PKA), cyclin-dependent kinase 5 (CDK5), C-Jun amino-terminal kinase (JNK), and calcium/calmodulin-dependent protein kinase II (CaMKII). These kinases have been shown to phosphorylate tau at more than 30 serine/threonine residues in AD [8,27].

Therapies targeting tau protein are extensively studied as a possible solution for AD (see Table 1), primarily focusing on the modulation of kinase/phosphatase systems. For example, the inhibition of GSK-3β activation by small molecules such as lithium chloride has been considered an attractive therapeutic approach to slow the progression of the disease [1,8]. Furthermore, PP2A activators such as sodium selenate and memantine are also used to modulate post-translational tau alterations. Other mechanisms involve inhibiting phosphodiesterase-4 (e.g., using the BPN14770 compound) and O-GlcNAcase enzymes (e.g., employing the MK-8719 compound), with the latter resulting in the suppressed deglycosylation of tau [8]. Moreover, microtubule stabilizers like davunetide and abeotaxane, as well as acetylation inhibitors such as salsalate, are currently being investigated as prospective disease-modifying drugs in AD. An alternative approach to reducing tauopathy involves the use of anti-tau antibodies (e.g., semorinemab, gosuranemab, zagotenemab, tilavonemab) and anti-tau vaccines (e.g., AADvac1, ACI-35), which are currently in clinical trials and appear to show promising efficacy in the treatment of AD [3,8].

Furthermore, recent studies have elucidated another potential therapy for Alzheimer’s disease, involving the inhibition of SIRT1, a histone deacetylase that regulates transcription factors. This enzyme has been found to down-regulate tau protein expression in AD by acting on transcription factor C/EBPα [28]. On the other hand, a novel therapy that uses antisense oligonucleotides (ASOs) and small interfering RNA (siRNA) is also being investigated as an alternative strategy to reduce tau expression [8]. Additional research indicates that a multi-targeted approach to treating Alzheimer’s disease may offer superior outcomes. This is exemplified by the potential of deoxyvasicinone analogs, which can function as dual inhibitors of acetylcholinesterase (AChE) and tau protein aggregation [29]. In addition to this compound, other molecules that have shown the ability to inhibit tau aggregation include derivatives of methylthioninium [8].

**Table 1:** Anti-tau therapeutic approach in Alzheimer’s disease.

Mechanisms of Tauopathy Reduction	Compounds	References
Phosphatase (PP2A) activators	Sodium selenate, memantine	[8]
Kinase (GSK-3β) inhibitors	Lithium chloride	[1,8]
Phosphodiesterase-4 inhibitors	BPN14770	[8]
Acetylation inhibitors	Salsalate	[3,8]
Tau aggregation inhibitors	Deoxyvasicinone analogs, methylthioninium derivatives	[8,29]
Tau deglycosylation inhibitors (GlcNAcase enzyme inhibitors)	MK-8719	[8]
Microtubule stabilizers	Davunetide, abeotaxane	[3]
Anti-tau vaccines	AADvac1, ACI-35	[3,8]
Anti-tau antibodies	Semorinemab, gosuranemab, zagotenemab, tilavonemab	[3,8]
Reducing tau expression	SIRT1 inhibitors ASOs, siRNA	[8,28]

## 4. Oxidative Stress

Oxidative damage occurs when the balance between oxidants and antioxidants is disturbed, resulting in an increased concentration of free radicals. These radicals are capable of inducing the oxidation of intracellular proteins, nucleic acids, and lipid membranes, thereby interfering with cellular functions and compromising membrane integrity. This cascade of events ultimately culminates in neuronal apoptosis [30,31]. Reactive oxygen species (ROS), along with reactive nitrogen species (RNS), act as the main progenitors of free radical species, with their enhanced accumulation evident in the preliminary stages preceding the onset of AD in affected persons. These ROS and RNS originate predominantly from mitochondrial glucose oxidation, involving molecular entities such as peroxynitrite (ONOO–), superoxide (O_2_–), hydrogen peroxide (H_2_O_2_), nitric oxide (NO–), and hydroxyl (OH–) [32]. Mitochondrial superoxide is the most reactive among these species, instigating a cascade of reactive molecules responsible for various pathological conditions, including, but not limited to, AD [33]. Reactive oxygen species have been identified as key contributors to cellular damage associated with aging and neurodegenerative disorders, including AD [34]. Regarding the factors that lead to increased ROS production, it is recognized that the accumulation of amyloid-beta can induce dysfunction in the endoplasmic reticulum (ER) and mitochondria, characterized by decreased activity and compromised membrane integrity, thus exacerbating ROS production [33,35]. Furthermore, a prominent anomaly within the mitochondrial electron transport chain in AD is characterized by a deficiency of cytochrome C oxidase, leading to the increased production of reactive oxygen species [36].

In AD and certain other age-related neurodegenerative diseases, the brain, which uses 20% of the body’s oxygen, is predisposed to oxidative damage [37]. This vulnerability comes from dysregulated glucose metabolism, mitochondrial impairment, and a compromised antioxidant defense system. The increased susceptibility of the brain to oxidative damage is further exacerbated by the abundance of polyunsaturated fatty acids and the presence of redox-active transition metal ions [38,39]. Furthermore, the brain has a restricted reservoir of antioxidants, making it more susceptible to damage induced by reactive oxygen species [40,41]. These antioxidant biomolecules include antioxidant defense enzymes such as superoxide dismutase (SOD), glutathione reductase (GR), catalase (CAT), thioredoxin, and glutathione peroxidase (GPx). Furthermore, there is a cellular mechanism that involves the activation of nuclear factor erythroid-2-related factor 2 (Nrf2), which leads to the up-regulation of these antioxidant enzymes [30,31]. Therefore, under conditions of excessive oxidative damage, NRF2 is translocated to the nucleus and associates with the antioxidant response element (ARE), thus initiating the transcription of genes responsible for antioxidant defense [42]. ROS are known to hinder the functionality of phosphatase 2A (PP2A), a critical regulator of processes that include signal transduction, cell cycle, cell differentiation, and transformation [43]. The inhibition of PP2A facilitates the stimulation of glycogen synthase kinase-3 beta (GSK-3β), an enzyme involved in tau protein phosphorylation. This activation may precipitate the hyperphosphorylation of tau proteins, culminating in the formation of neurofibrillary tangles in the brains of individuals with Alzheimer’s disease, thus aggravating the pathogenesis of AD [32,44]. In addition, oxidative stress can intensify the pathophysiological characteristics of AD by activating the c-Jun N-terminal kinase (JNK) and AMP-activated protein kinase (AMPK) pathways, which are involved in the regulation of tau phosphorylation and may also contribute to the accumulation of neurofibrillary tangles [45,46].

In recent studies, antioxidants have gained substantial interest as potential therapeutic agents. A wide range of phytochemicals, including compounds from ginseng root, flavonoids, terpenoids, chalcones, polyphenols, and alkaloids, have shown the ability to activate NRF2 and even stimulate the expression of endogenous antioxidant enzymes, including superoxide dismutase, catalase, and glutathione (GSH) [42]. Therefore, a multitude of molecules have been found to exhibit antioxidant properties, functioning not only as activators of Nrf2, such as sulforaphane and hydralazine (the latter also inducing autophagy), but also as mimics of glutathione peroxidase, exemplified by ebselen [8,47]. Consequently, the modulation of the NRF2 signaling pathway has considerable potential to prevent or delay the onset and progression of AD by regulating various pathways, such as heme oxygenase-1 (HO-1), phase II antioxidant enzymes, the antioxidant response element (ARE), beta-site amyloid precursor protein cleaving enzyme 1, and extracellular signal-regulated kinases (ERK), as evidenced in both animal and cellular models [42]. In addition, recent research highlights the potential of various additional molecules for antioxidant therapy in AD. These encompass natural antioxidants like resveratrol, alpha-tocopherol, rosmarinic acid, curcumin, and quercetin, alongside several exogenous antioxidants such as luteolin, melatonin, edaravone, coenzyme Q10, epigallocatechin-3-gallate (EGCG), berberine, and hyperoside [8].

### 4.1. Advanced Lipid Peroxidation Products

Lipid peroxidation is a significant form of oxidative injury, with 4-hydroxy-2-nonenal (HNE) serving as the main marker [24,48]. This highly reactive neurotoxic by-product of lipid peroxidation exerts a substantial impact on the pathophysiology, biochemical characteristics, and clinical symptoms of Alzheimer’s disease and its prodromal stages [48]. One study indicated that the hippocampus of patients with AD exhibits elevated concentrations of HNE-histidine Michael adducts. This covalent modification of the histidine side chain in amyloid-beta may be associated with an enhanced aggregation of tau protein [49]. Thus, HNE is notable for its ability to form covalent bonds with critical proteins located in neuronal membranes, mitochondria, and the cytosol, thus compromising its structural integrity. The covalent attachment of HNE to these vital neuronal proteins triggers a sequence of functional impairments in the proteins, culminating in neuronal apoptosis. In addition, HNE affects glucose metabolism, leading to a decrease in ATP production. This alteration, combined with the modification of ion motive ATPases by HNE and Ca^2+^ signaling disruption, also contributes to neuronal cell death [48]. Furthermore, Tamagno et al. [50] showed that increased lipid peroxidation instigates the activation of BACE 1, which leads to the increased synthesis of amyloid-beta.

### 4.2. Advanced Glycation End Products (AGEs)

AGEs result from the non-enzymatic reaction between reducing sugar and proteins or lipids, leading to their accumulation under conditions marked by elevated glucose levels [51]. AGE synthesis has been shown to be associated with modified immunogenicity, reduced ligand binding, increased free radical activity, and altered protein half-life, leading to neurodegenerative disorders [52,53]. Thus, the interaction of AGEs with the receptor for AGEs (RAGE) instigates the synthesis of free radicals and pro-inflammatory cytokines. Meanwhile, the interaction among AGEs, tau protein, and amyloid-beta has been established to influence neuronal functionality [51]. Furthermore, inverse relationships between AGE concentrations and cognitive performance have been documented [54].

Moreover, elevated concentrations of AGEs have been correlated not only with the onset and intensity of paratonia, but also with the decreased mobility observed in the early phases of AD [51,55]. As a consequence, the early identification of the disorder may facilitate the implementation of intervention strategies, encompassing physical activity regimens and nutritional guidance, potentially in conjunction with pharmacological approaches. Such multifaceted interventions aim to mitigate functional deterioration, thus prolonging the period of autonomy of people with dementia [51]. Recently, a new molecule, called azeliragon, has been identified as a prospective therapy for AD. It acts as a RAGE inhibitor, thus reducing microglial activation. Consequently, it exhibits both anti-inflammatory and anti-amyloid-beta effects, resulting in decreased Aβ plaque formation [56].

## 5. Mitochondrial Dysfunction

Numerous studies suggest that compromised mitochondrial integrity may significantly impact the etiopathogenesis of AD. Consequently, investigating the diverse mitochondrial mechanisms involved in the pathogenesis of AD emerges as a potential avenue for the identification of novel therapeutic targets for this condition [57].

Tau pathology in Alzheimer’s disease is intricately linked with mitochondrial changes. The hyperphosphorylation and aggregation of tau proteins are associated with disruptions in mitochondrial dynamics, particularly affecting axonal transport and bioenergetics. These alterations encompass anomalies in mitochondrial localization within neurons, imbalances in mitochondrial fusion and fission mechanisms, and deviations in ATP production. Consequently, an increased generation of reactive oxygen species occurs, along with mitochondrial depolarization. Such aberrations in tau phosphorylation and aggregation contribute to neuronal and synaptic deterioration, culminating in the cognitive decline that is typical for Alzheimer’s disease [58].

The aggregation of amyloid-beta within the brain mitochondria of Alzheimer’s disease patients also leads to altered mitochondrial morphology, impaired respiratory function, decreased release of adenosine triphosphate (ATP), compromised mitochondrial dynamics, and increased mitochondrial oxidative stress [24]. Mitochondrial dynamics is critically intertwined with mitochondrial function. Dysfunctions in the processes of mitochondrial fusion and fission influence mitochondrial size, morphology, and quantity. Furthermore, modifications in mitochondrial breakdown mechanisms (via mitophagy or macroautophagy) and biosynthesis significantly impact mitochondrial function and integrity. Various models of Alzheimer’s disease have revealed perturbations in mitochondrial dynamics, affecting not only neuronal cells but also astrocytes [59]. In the context of AD, appoptosin overexpression can trigger the activation of the intrinsic caspase pathway. In particular, a reduction in appoptosin expression has been found to confer protection against the neurotoxic effects of amyloid-beta [24].

Contemporary research has elucidated that in brain tissue from patients with AD, the activity of mitochondrial ATP synthase is compromised. This impairment has been attributed to the diminution of the oligomycin-sensitive conferring protein subunit, as well as potential alterations in the O-GlcNAcylation of the ATP synthase subunit α [57]. Amyloid-ß-binding alcohol dehydrogenase (ABAD) and cyclophilin D (CypD) constitute additional mitochondrial proteins that have been identified to contribute to mitochondrial dysfunction [24].

In alignment with the dysregulated energy metabolism characteristic of Alzheimer’s disease, successive gene expression analyses have consistently pinpointed anomalies in the metabolic pathways associated with mitochondria. This accumulation of evidence supports the presence of compromised bioenergetic systems within mitochondria in the context of AD [57]. Thus, using gene set enrichment analysis (GSEA), it has been elucidated that the disruption of mitochondrial import routes and the inhibition of mitochondrial oxidative phosphorylation (OXPHOS) constitute distinguishing features of Alzheimer’s disease [60]. Furthermore, a bioinformatic examination of four transcriptomic data sets related to the hippocampus of patients with AD identified the oxidative phosphorylation pathway as one of the most prominent pathways involved in the pathophysiology of the disease [57]. In addition, an analysis that uses microarray techniques complemented by quantitative reverse transcription polymerase chain reaction (RT-PCR) methodologies revealed a notable down-regulation of 15 of the 51 components involved in the oxidative phosphorylation, the tricarboxylic acid (TCA) cycle, the glycolytic pathway and their associated metabolic pathways, in the context of Alzheimer’s disease [61].

Recently, a connection between mitochondrial DNA (mtDNA) variations and Alzheimer’s disease has been demonstrated, indicating that mtDNA polymorphisms and haplogroups may influence its development. Furthermore, the pattern of maternal inheritance of mtDNA corresponds to the more frequent maternal transmission of AD in families with a history of the disease, underscoring the potential role of mtDNA in the pathology of AD [57].

A therapeutic approach that directly addresses mitochondrial function involves the modulation of mitochondrial activities, particularly those pertaining to bioenergetics. For example, oxaloacetate (OAA), an intermediary compound involved in both gluconeogenesis and the Krebs cycle, has been observed to increase bioenergetic fluxes and elevate certain parameters related to the bioenergetic infrastructure of the brain [62]. Furthermore, nicotinamide adenine dinucleotide (NAD), a crucial intermediary in multiple mitochondrial metabolic pathways that include oxidative phosphorylation, the cycle of tricarboxylic acid (TCA), and glycolysis, is currently being investigated as a novel approach for AD [63]. Recent investigations have also elucidated that the moderate attenuation of complex I activity at the FMN subunit, specifically NDUFA1, facilitated by the CP2 compound, results in enhanced respiratory capacity and a reduction in proton leakage. This compound, CP2, has shown potential to improve cognitive and pathological impairments in various animal models of Alzheimer’s disease and is in the process of development for the treatment of AD [64]. However, focusing on mitochondrial proteins, particularly the fission protein dynamin-related protein 1 (Drp1), also emerges as a possible therapeutic strategy for AD. The interaction between Drp, hyperphosphorylated tau, and amyloid-beta impacts mitochondrial morphology, movement, and energy generation, resulting in alterations of ATP production. Thus, decreased Drp1 GTPase activity has demonstrated neuroprotective outcomes in experimental models of AD, suggesting that targeting Drp1 may offer a promising route to alleviate AD-related neurodegeneration [65].

## 6. Neuroinflammation

Neuroinflammation is increasingly recognized as a significant contributor to the pathogenesis of Alzheimer’s disease, with multiple underlying mechanisms involved in its progression [66,67]. Furthermore, the clear involvement of neuroinflammation in AD may be supported by the discovery of a significant accumulation of inflammatory mediators surrounding amyloid plaques and neurofibrillary tangles [67]. Thus, certain inflammatory cytokines can induce an increased amyloid-beta deposition by up-regulating both the expression of beta-site APP cleavage enzyme 1 and the amyloid precursor protein. Furthermore, key cytokines, including tumor necrosis factor-α (TNF-α), interleukin-6 (IL-6), and interleukin-1β (IL-1β), have been identified as contributors to tau phosphorylation, thus accelerating the formation of neurofibrillary tangles, which culminate in the symptomatic manifestation of AD [66]. Targeting neuroinflammation through reductions in the levels of these cytokines can be achieved by inhibiting JNK signaling, which plays an important role in Alzheimer’s disease by regulating Aβ production [68,69].

Another important pathway modulating neuroinflammation in AD is the p38 MAPK pathway, which may play a major role in exacerbating cognitive deterioration by up-regulating certain cytokines, such as IL-1β and TNF-α, in addition to activating the NF-κB pathway. Consequently, p38 MAPK is recognized as a promising molecular target for innovative therapies in Alzheimer’s disease. Thus, the administration of a p38 MAPK inhibitor in transgenic mice has resulted in a reduction in neuroinflammatory states and amyloid-beta accumulations, thus improving spatial memory performance [70].

Other studies are focused on natural solutions for anti-inflammatory therapy in Alzheimer’s disease. For instance, sodium oligomannate, a marine-derived oligosaccharide, may have demonstrated beneficial effects in mild to moderate forms of the disease, acting not only by reducing neuroinflammation but also by contributing to gut–brain homeostasis. Some studies even suggest that its efficacy may surpass that of donepezil, with a favorable safety profile [71,72].

In recent times, there has been significant growth in the utilization of nanoparticle-based anti-inflammatory therapy in AD. More of these molecules show not only the capability to enhance drug transport across the blood–brain barrier, but also intrinsic anti-inflammatory and anti-Aβ effects. Several examples include lipid-based nanoparticles, selenium, gold, nanoemulsions, and ceric dioxide [7]. Furthermore, neural stem cell transplantation (NSC) is emerging as a promising therapy for AD, primarily operating through the regulation of neuroplasticity through BDNF, while also reducing neuroinflammation and the aggregation of tau and Aβ [73]. Other drugs provide a multitargeted approach in Alzheimer’s disease, affecting multiple mechanisms. For instance, edaravone may be effective in mice by reducing not only neuroinflammation, but also oxidative stress, tau hyperphosphorylation, Aβ accumulation, synaptic impairment, and neuronal degeneration [74].

Microglia and astrocytes constitute the main components of the brain immune system and play a crucial role in the process of neuroinflammation [67].

### 6.1. Microglia

Microglia serve as resident phagocytes within the brain, mainly tasked with the clearance of apoptotic or necrotic cells and the removal of unfolded or misfolded proteins [75]. As immune cells of the central nervous system, microglia are sensitive to pathogenic challenges and cellular degeneration and play a direct role in the synaptic and cellular loss observed in Alzheimer’s disease [76]. A central histopathological feature of Alzheimer’s disease is the development of amyloid-beta plaques, which precipitates the elevated expression of pro-inflammatory molecules. This event is concomitant with a metabolic shift in microglia, transitioning from oxidative phosphorylation to aerobic glycolysis. This metabolic alteration is closely related to the secretion of cytokines and compromised phagocytic efficacy in microglial cells [77].

The framework of microglial polarization along a spectrum of pro-inflammatory to regulatory phenotypes remains relevant for discussing microglial roles in AD. However, recent advances in transcriptomic analyses have revealed multiple intermediate phenotypes that exist beyond the traditional binary classification, highlighting the complexity and adaptability of microglial responses. The pro-inflammatory activation pathway in microglia is associated with the induction of inflammatory responses and neurotoxic effects [78]. In this activated state, microglia are characterized by the release of pro-inflammatory cytokines and chemokines, including the tumor necrosis factor-alpha (TNF-α), interleukin-1 beta (IL-1β), interleukin-12 (IL-12), interleukin-6 (IL-6), interferon-gamma (IFN-γ), and the motif C-C of the chemokine ligand 2 (CCL2) [78,79,80]. Furthermore, microglia with a pro-inflammatory phenotype express inducible nitric oxide synthase (iNOS), which catalyzes the conversion of arginine to nitric oxide (NO), contributing to inflammatory responses in the neural environment. The resulting accumulation of NO exacerbates the neurotoxic effects of glutamate and leads to neuronal damage [78]. In contrast, microglia with a regulatory phenotype adopt an anti-inflammatory profile, characterized by the production of interleukins such as IL-10, IL-13, IL-4, and transforming growth factor-beta (TGF-β) [78,79]. This kind of microglia also express arginase 1, which converts arginine to polyamines, modulating inflammation and facilitating tissue repair [78]. Therefore, microglial cells play a dual role in AD, functioning as both beneficial and detrimental entities. In the initial stages of the disease, microglial activation is associated with neuroprotective effects, such as the suppression of beta-amyloid hyperproduction, the release of neurotrophic factors, and the clearance and phagocytosis of cellular debris and necrotic cells [81].

In AD, the dysregulation of microglial activation, with a bias toward a pro-inflammatory phenotype, contributes to persistent neuroinflammation, thereby exacerbating neuronal damage and accelerating disease progression. The Notch signaling pathway, a highly conserved cellular communication mechanism, may play a role in this dysregulation, potentially emerging as a therapeutic target to restore balance in microglial states and alleviate neuroinflammation in AD. Furthermore, emerging studies have suggested that Notch signaling could directly influence AD pathology by affecting both the production and clearance of amyloid-beta plaques [78].

In the advanced phases of AD, a notable decline has been demonstrated in microglial phagocytic activity concerning amyloid-beta plaques. Considering that microglial cells are the principal amyloid-beta eliminators in the brain, this deficiency leads to the accumulation of these plaques, exacerbating the progression of AD [75,78]. On the other hand, the microglial hyperphagocytosis of normal neuronal synapses emerges as a crucial factor that contributes to cognitive decline in Alzheimer’s disease, indicating the complex and critical role of microglial function in the pathophysiology of the disease [78]. 

Activated microglia have also been identified as contributors to tau pathology, exerting their influence directly through the induction of neuroinflammation or indirectly by disrupting neuronal homeostasis. Recent research has shown that microglia colocalize with neurofibrillary tangles in postmortem brain tissue from patients with Alzheimer’s disease, and aggregated tau is internalized by these cells in vivo and in vitro [80]. Furthermore, an autopsy study examining the temporal neocortex of 15 control subjects without dementia and 91 AD patients revealed that in the postmortem brains of AD patients, even after the cessation of Aβ plaque growth, there is a linear and positive correlation between increased microglial density and the burden of neurofibrillary tangles [82]. The CX3C chemokine receptor 1 (CX3CR1) has been identified to play a pivotal role in microglia-mediated tau pathology. In particular, the hippocampus and frontal cortex of the brains with AD exhibit significantly reduced levels of CX3C chemokine ligand 1 (CX3CL1) and CX3CR1 compared to controls, indicating that signaling through the CX3CL1/CX3CR1 pathway is compromised in AD [80].

Genome-wide association studies (GWASs) have pinpointed risk loci that exhibit a strong correlation with the pathogenesis of AD. Many of these loci are located near or within genes that are expressed primarily in microglial cells, suggesting a significant genetic contribution of microglia to the development and progression of AD [78]. Thus, in the context of late-onset Alzheimer’s disease (LOAD), CD33 was identified as one of the initial genes associated with the disease through comprehensive genome-wide association studies [83]. Postmortem examinations have revealed an up-regulation of CD33 expression in microglial cells in the cerebral tissues of individuals with Alzheimer’s disease. This increased expression exhibits a correlation with both the severity of amyloid-beta accumulation and the extent of cognitive deterioration [78]. The CD33M isoform hinders the phagocytic activity of microglia toward the Aβ42 peptide, which is recognized as the amyloid-beta form most prone to aggregation. Consequently, low levels of this particular isoform are correlated with increased clearance of the Aβ42 peptide [83]. Therefore, new therapeutic strategies in Alzheimer’s disease aim to inhibit CD33 activity to reverse the altered microglial phagocytic function of microglia concerning amyloid-beta [84].

An additional gene associated with the risk of AD is TREM2 (Triggering Receptor Expressed on Myeloid Cells 2), which plays a pivotal role in facilitating the phagocytic activity of microglia specific to Aβ [77,84]. Recent research has elucidated that TREM2 facilitates the microglial response to amyloid-beta through independent and spleen tyrosine kinase (SYK)-dependent mechanisms, as evidenced in the 5xFAD model of Alzheimer’s disease [84]. Thus, a TREM2 deficiency has been shown to result in compromised microglial survival, migration, and phagocytic capabilities [82]. The soluble variant of TREM2 (sTREM2) has emerged as a valuable biomarker for AD pathology and cognitive degeneration, as sTREM2 levels in cerebrospinal fluid (CSF) increase during the early symptomatic phase of Alzheimer’s disease [78,84]. In particular, the up-regulation of TREM2 expression, achieved through the overexpression of human TREM2, has demonstrated efficacy in reducing amyloid plaque deposition. Furthermore, the use of agonist antibodies targeting TREM2 has yielded promising results, evidenced by a reduction in Aβ burden and improvements in behavioral performance [78].

Recently, a distinct subset of microglia, called disease-associated microglia (DAM), has been identified [78]. DAM cells are characterized by altered gene expression profiles [78], with the down-regulation of genes associated with microglial homeostasis (P2RY13, CX3CR1, P2RY12) and the up-regulation of genes linked to AD risk factors, including TREM2 and ApoE [76,77,84]. Disease-associated microglia are known to undergo a sequential two-step activation process, characterized by TREM2-independent and -dependent pathways. The initial activation phase encompasses a TREM2-independent down-regulation of homeostatic markers, including P2ry12, Cx3cr1, and Tmem119. The subsequent activation stage is dependent on TREM2 and is associated with the increased expression of genes involved in lipid metabolism and phagocytic activity [84]. Signaling pathways leading to microglial activation, including mitogen-activated protein kinases (MAPKs), toll-like receptors (TLRs), janus kinase/signal transducer and transcription activator (JAK/STAT), nuclear factor kappa light chain enhancer of activated B cells (NF-κB), and phosphoinositide 3-kinases/AKT (PI3K/AKT), may contribute to the development of precise therapeutic strategies targeting Alzheimer’s disease [78].

Inflammasomes, which make up a collection of polyprotein complexes present within microglial cells [85], have been shown to play a significant role in the pathology of Alzheimer’s disease, with a specific emphasis on the NLRP3 inflammasome [77]. Consequently, it has been shown that activation of the NLRP3 inflammasome can be triggered by a variety of stimuli, including oxidative stress, amyloid-beta, phosphorylated tau, and aggregated tau proteins, ultimately resulting in neuroinflammation and neuronal damage [77,85]. The connection between microglial cell metabolism and the activation of the NLRP3 inflammasome, primarily through glycolytic pathways, may hold significance for therapeutic approaches in AD. The inhibition of these pathways reduces neuroinflammation and degeneration, suggesting that targeting these mechanisms could be beneficial in the treatment of AD [77]. Thus, the inhibition of NLRP3 in TauP301S transgenic mice has been found to decrease tau phosphorylation and Aβ accumulation in the hippocampus [85].

The peroxisome proliferator-activated receptor-gamma (PPAR-γ), a ligand-inducible transcription factor that forms part of the nuclear receptor superfamily, exhibits notable expression in microglial cells and plays a critical role in the pathogenesis of Alzheimer’s disease [78]. Its regulatory role within microglial cells encompasses the modulation of pro-inflammatory and anti-inflammatory cytokines, with its activation having therapeutic potential for AD [78,84]. Consequently, the compound pioglitazone, recognized as a PPAR-γ agonist, has been shown to inhibit the production of pro-inflammatory cytokines in lipopolysaccharide (LPS)-activated rat microglial cell lines [84]. On the other hand, the antagonism of PPAR-γ promotes a shift in microglial activation toward a more regulatory and reparative phenotype, primarily by enhancing autophagy through the liver kinase B1/AMP-activated protein kinase (LKB1/AMPK) signaling pathway. Taking into account the association of impaired autophagy with the accumulation of amyloid-beta and tau proteins, the participation of PPAR-γ in the regulation of autophagy further highlights its potential as a therapeutic target in Alzheimer’s disease [78].

Another important molecule with high expression in AD brain microglial cells is receptor-interacting protein kinase 1 (RIPK1), which contributes to the TNF-α-induced necroptosis pathway. The inhibition of RIPK1 may facilitate the degradation of amyloid-beta by microglia, resulting in reduced levels of inflammatory mediators, thus improving cognitive deficits in transgenic mice expressing mutant amyloid precursor protein and presenilin 1 (APP/PS1) [86]. Recently, the elevated expression of the calcium homeostasis modulator family protein type 2 (Calhm2) has been noted in microglia within the AD model. Consequently, the inhibition of Calhm2, known for its role in the regulation of calcium influx, can reduce neuroinflammation and the accumulation of Aβ proteins, culminating in improved cognitive function [87]. As previously described, microglia offer a wide array of potential therapeutic approaches in Alzheimer’s disease (see Table 2), thus representing a crucial area of interest.

### 6.2. Astrocytes

**Figure 2:**
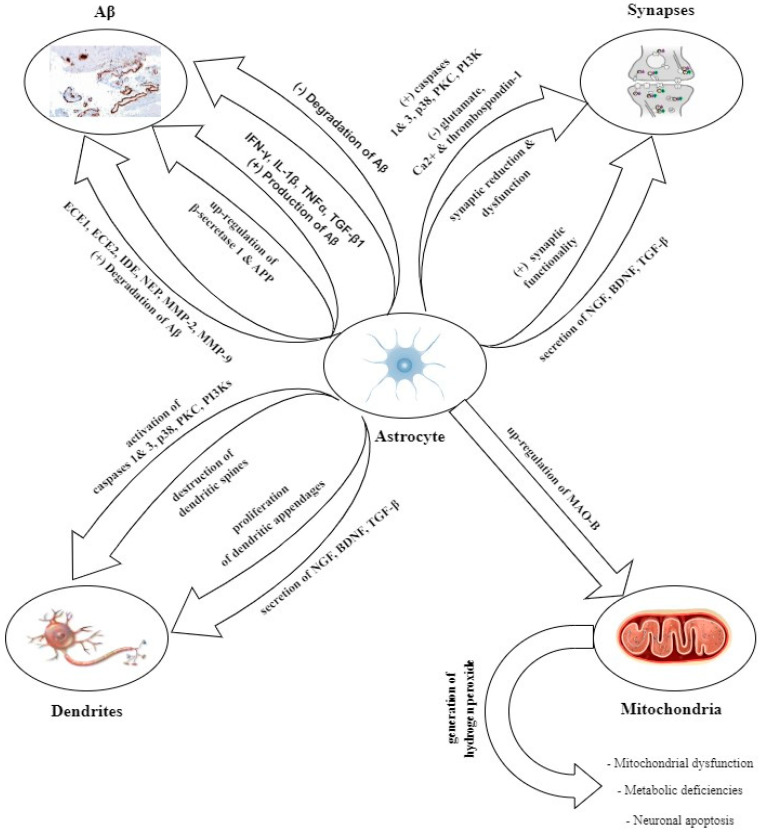
The effects of astrocytes on amyloid-beta deposition, dendrites, synapses, and mitochondria in Alzheimer’s disease. PKC: protein kinase C; PI3Ks: phosphoinositide 3-kinases; NGF: Neuron Growth Factor; BDNF: brain-derived neurotrophic factor; TGF-β: Tumor Beta Growth Factor; APP: amyloid precursor protein; IFN-γ: interferon-gamma; IL-1β: interleukin-1-beta; TNFα: Tumor Necrosis Factor alpha; Aβ: amyloid-beta; MMP-2; MMP-9: matrix metalloproteinases 2 and 9; ECE1; ECE2: endothelin converting enzymes 1 and 2; IDE: insulin-degrading enzyme; NEP: neprilysin; MAO-B: monoamine oxidase B, (+): stimulation, (-): inhibition.

Astrocytes appear to play a critical role in the onset and progression of Alzheimer’s disease through various mechanisms (see Figure 2), especially in its later stages [88]. These cells exhibit a dualistic nature, functioning in both pro-inflammatory and anti-inflammatory capacities [9,88]. Astrocytes with a pro-inflammatory phenotype are characterized by the secretion and production of a wide range of inflammatory factors and neurotoxins. In contrast, astrocytes with a regulatory phenotype are involved in the production of neurotrophic substances and play a supportive role in neuronal growth [9]. The interaction of amyloid-beta peptides with specific receptors in astrocytes has been shown to induce a shift in their functional dynamics from anti-inflammatory to pro-inflammatory [88].

In the context of AD, astrocytes undergo significant alterations in morphology, transcriptional profiles, and functional capacities [80]. Recent research has substantiated that pronounced reactive astrogliosis constitutes a characteristic morphological trait in the brains of Alzheimer’s disease mouse models and AD patients, which can precede the formation of amyloid-beta plaques [80,88,89]. Morphological analyses of postmortem brain samples from patients with Alzheimer’s disease have revealed a significant interaction between astrocytes and amyloid-beta deposits [88]. Additionally, the transcriptome analysis of postmortem brains from patients with AD has revealed that astrocytes exhibit alterations in glial gene expression, which correspond to the levels of amyloid and phosphorylated tau in the tissue. Indeed, astrocyte-mediated tauopathy risk loci, which encompass genes related to clustering, Myocyte Enhancer Factor 2C, and IQ Domain-Containing Protein K, have been identified in the postmortem brains of AD patients through single-nuclei RNA-sequencing transcriptomics [90].

Numerous studies have demonstrated the participation of astrocytes in the clearance of amyloid-beta in vitro, further highlighting their role in the attenuation of neurodegenerative processes associated with Alzheimer’s disease [80]. Thus, they are capable of synthesizing amyloid-beta-degrading proteases, such as endothelin converting enzymes 1 and 2 (ECE1 and ECE2), insulin-degrading enzyme (IDE), and neprilysin (NEP), which play a key role in the breakdown of monomeric amyloid-beta species [80,91]. Furthermore, they express the matrix metalloproteinases MMP-2 and MMP-9, which are involved in the degradation of fibrillar and monomeric forms of amyloid-beta [91]. On the other hand, AD conditions can also transform astrocytes into Aβ producers, as they exhibit the up-regulation of BACE-1 and APP in a neuropathological context. Meanwhile, agents such as IFN-γ, IL-1β, TNFα, and TGF-β1 stimulate astrocytes to synthesize Aβ [80,88]. Furthermore, astrocytes can engulf substantial amounts of partially digested Aβ protofibrils, leading to a decrease in their degradation capacity and contributing to neuronal apoptosis, as demonstrated in vitro and in vivo studies [80].

Within the context of Alzheimer’s disease, astrocytes are known to bidirectionally modulate synaptic functionality. From one perspective, astrocytes contribute to synaptic reduction both directly and indirectly. Therefore, they exhibit a direct impairment in phagocytic capacity, a condition influenced by the expression levels of ApoE4, C3, MERTK, and MEGF10.

On the other hand, decreased glutamate transport and signaling, together with disrupted Ca^2+^ signaling and the reduced expression of thrombospondin-1, collectively create a synaptotoxic environment that indirectly reduces synaptic density [92]. Through the activation of various mediators, such as caspases 1 and 3, p38, and protein kinase C (PKC), as well as pathways such as phosphoinositide 3-kinases, astrocytes can cause neuronal damage, the destruction of dendritic spines, and synaptic dysfunction, which ultimately results in cognitive impairment [88]. Contradictory, astrocytes facilitate dendritic appendage proliferation and enhance synaptic functionality through the secretion of neurotrophic factors, including the Neuron Growth Factor (NGF), brain-derived neurotrophic factor (BDNF), and Tumor Beta Growth Factor (TGF-β) [93]. Another mechanism by which astrocytes are involved in the development of AD involves the up-regulation of monoamine oxidase B (MAO-B) in these cells, culminating in an increased generation of hydrogen peroxide. This can potentially lead to neuronal harm and apoptosis, mitochondrial dysfunction, and metabolic deficiencies [88].

Regarding novel therapeutic possibilities based on astrocytes, identifying an effective approach to mitigate the pro-inflammatory phenotype of astrocytes may represent a promising strategy to slow the progression of AD. Furthermore, the application of selective inhibitors targeting specific astrocyte subtypes could help correlate distinct astroglial states with different phases of AD [88]. Furthermore, pharmaceuticals with anti-diabetic properties, particularly those capable of improving astrocyte metabolism, have therapeutic potential in the early stages of AD [94].

## 7. Insulin Resistance and Impaired Insulin Signaling

Alzheimer’s disease exhibits such a significant correlation with diabetes that it is often referred to as ‘type 3 diabetes’ [95]. Furthermore, people with type 2 diabetes mellitus have a higher risk, up to 65%, of developing Alzheimer’s disease compared to those without diabetes [96]. It is known that the initial phases of Alzheimer’s disease are characterized by reduced glucose uptake and inadequate energy metabolism [97]. Meanwhile, a decline in cerebral glucose metabolism has been documented to occur more than a decade before the emergence of clinical symptoms associated with AD [95]. Cerebral insulin is recognized to facilitate the modulation of amyloid-beta clearance, oxidative stress, tau phosphorylation, lipid metabolism, cerebral blood flow, and memory genesis [98]. Consequently, any changes in insulin signaling within the brain may play a pivotal role in the pathogenesis of AD [97,98]. On the other hand, the literature presents divergent results on the neurodefensive properties of the insulin signaling pathway. Notably, it has been observed that diminished neuronal insulin signaling may positively influence lifespan regulation and potentially delay the onset of age-related degenerative processes [99].

When entering the brain, insulin undergoes degradation by the insulin-degrading enzyme (IDE), which also possesses the ability to degrade the amyloid-beta protein. Significantly, insulin and Aβ engage in a competitive interaction for IDE binding, with insulin showing a higher affinity relative to Aβ. Consequently, under conditions of hyperinsulinemia, the preferential binding of insulin to IDE results in the accumulation of the Aβ protein [100,101]. Fascinatingly, amyloid-beta adheres and impedes the binding of insulin-to-insulin receptors (IRs), thus exacerbating insulin resistance [99]. The binding of insulin to its receptors in the brain initiates gene expression through the mitogen-activated protein kinase (MAP kinase) and Akt/protein kinase B (PKB) signaling pathways. Through these mechanisms, insulin facilitates improved glucose uptake, protein synthesis, autophagy, and mitochondrial functionality, while simultaneously inhibiting apoptosis [102]. Therefore, by inhibiting AMP-activated protein kinase, peripheral hyperinsulinemia is responsible for sustained inflammatory processes, which may serve as mediators in the association between type 2 diabetes mellitus (T2DM) and Alzheimer’s disease [99]. Studies have shown that insulin resistance is also associated with tau hyperphosphorylation [103].

Insulin can prevent tau phosphorylation by acting as an inhibitor of GSK3β, the enzyme responsible for facilitating tau phosphorylation. Consequently, it is hypothesized that in states of insulin resistance, GSK3β activity is increased, resulting in increased tau phosphorylation and the subsequent formation of NFTs [104,105]. In addition, insulin resistance is associated with a reduction in the acetylcholine levels within the brain, leading to disturbances in cholinergic systems, which play an important role in the pathophysiology of Alzheimer’s disease [99].

Recent research has increasingly focused on exploring the link between insulin signaling and AD, demonstrating cognitive improvements in patients with minor cognitive dysfunction and AD, following the use of antihyperglycemic agents including metformin, intranasal insulin, incretins, and thiazolidinediones. Furthermore, the efficacy of intranasal insulin appears to be influenced by the individual’s ApoE genotype status, while no such improvement has been observed in patients with APOE4 [106].

## 8. The Role of Cholinesterases (Acetylcholinesterase and Butyrylcholinesterase) in Alzheimer’s Disease

Cognitive dysfunction in Alzheimer’s disease and adult-onset dementia disorders is markedly affected by the dynamics of cholinergic neurotransmission [107]. The cholinergic hypothesis theorizes that the pathogenesis of Alzheimer’s disease is primarily attributed to a decrease in the specific subtypes of acetylcholine receptors (AChs), some structural modifications in cholinergic synapses, or the death of neurons that produce ACh, and consequently, the impairment of cholinergic neurotransmission [108]. In the brain, two cholinesterase enzymes, namely acetylcholinesterase (AChE) and butyrylcholinesterase (BChE), are responsible for the hydrolytic degradation of acetylcholine [107]. In individuals with Alzheimer’s disease, acetylcholinesterase exhibits a higher prevalence than butyrylcholinesterase within cerebral tissues, playing a pivotal role in the degradation of acetylcholine in the cerebral cortex and hippocampus. Research indicates a notable reduction in AChE activity by 67% relative to the baseline in the temporal lobe and hippocampus throughout the progression of AD. Concurrently, an increase in BChE activity, reaching up to 165% of standard levels, has been observed [109]. BChE is predominantly expressed in white matter and glial cells (specifically astrocytes), while AChE is localized in neuronal areas that are crucial for cognition and behavior, regions that undergo functional impairment in Alzheimer’s disease. Moreover, the presence of BChE in amyloid plaques and neurofibrillary tangles suggests a potential contributory role of this protein to the etiopathogenesis of AD [110].

Inhibitors of acetylcholinesterase/cholinesterase, which represent the primary category of pharmacological agents currently employed in the therapeutic management of AD, are incapable of fully arresting the progression of Alzheimer’s disease. Moreover, in light of the wide range of adverse reactions linked to existing cholinesterase inhibitors, it becomes imperative to forge new therapeutic agents [107].

## 9. The Serotoninergic System

The impairment of dopaminergic, serotoninergic, and cholinergic neurons within the diffuse modulatory nervous system is increasingly recognized to play a substantial role in the pathophysiology associated with Alzheimer’s disease [111,112].

Concerning the role of serotonin in the pathogenesis of Alzheimer’s disease, it is acknowledged that abnormal levels of serotonin (5-HT) lead to decreased signaling through the Aβ pathway, thus facilitating the accumulation of Aβ in the brain [113]. Furthermore, lesions observed in the raphe nucleus during the initial stages of Alzheimer’s disease further suggest a link between serotonin dysregulation and the onset of this neurodegenerative condition. Age-associated mitochondrial dysfunction has been postulated to serve as an initiating factor in raphe neuronal degeneration, potentially leading to a decrease in serotonin function in elderly individuals at risk for Alzheimer’s disease [114,115]. Moreover, the altered functionality of mitochondrial enzymes, such as monoamine oxidase, is thought to contribute to the dysregulation of serotonin observed in Alzheimer’s disease. However, increasing evidence indicates that both serotonin dysregulation and mitochondrial dysfunction are key contributors to inflammatory neuronal damage [111].

VMAT2 is the primary protein responsible for serotonin uptake in synaptic vesicles, allowing its storage and eventual release [116]. The function of VMAT2 is driven by the energy-consuming V-type H+-ATPase and is modulated by phosphorylation, both being ATP-dependent mechanisms [117,118,119]. Furthermore, the process of synaptic-vesicular transport is characterized by a high demand for energy [120]. Given the critical role of mitochondria as the main source of ATP in the presynaptic region, it is reasonable to infer that the generation of mitochondrial ATP is essential for the effective storage and transport of serotonin [121]. Furthermore, any disruption in mitochondrial bioenergetics could also affect serotonin recycling and reuptake processes, considering that the efficacy of the serotonin transporter (SERT) is intricately linked to the maintenance of a Na^+^/K^+^ gradient across the cell membrane, a process facilitated by energy-demanding Na^+^/K^+^ ATPase pumps [111,122].

The regulatory role of mitochondria in calcium (Ca^2+^) homeostasis may also play a significant role in serotonin neurotransmission. Compromised mitochondrial capacity to retain Ca^2+^ could potentially hinder serotonin regulation, affecting its transmission efficiency [111].

Recently, preclinical studies have shown that the contribution of serotonin to cognitive decline and neuropathological features associated with Alzheimer’s disease could be indirectly influenced through the microbiota–gut–brain axis, thus affecting not only the development but also the onset of the disease. Consequently, the potential of fecal microbiota transplantation, together with the administration of pre- and probiotics and dietary modifications, in modulating the brain’s serotonergic neurotransmitter system, which originates in the gastrointestinal tract, has attracted substantial interest [123].

The regulation of serotonin and its receptor functions using 5-HT receptor agonists and antagonists may also offer potential therapeutic advantages in the pathogenesis of Alzheimer’s disease [9,123,124]. Furthermore, in their research, Cirrito et al. [125] established that the acute administration of selective serotonin reuptake inhibitors (SSRIs) effectively reduced the synthesis of neurotoxic amyloid-beta proteins, which play a central role in the pathology of Alzheimer’s disease. This was observed in murine models exhibiting the overexpression of the amyloid protein precursor/presenilin-1 gene (APP/PS1) [125].

## 10. The Microbiota–Gut–Brain Axis

The gut–brain axis encompasses a complex interplay involving immune, endocrine, neural, and metabolic pathways, contributing to the maintenance of cerebral homeostasis [126]. Recent studies on the microbiota–gut–brain axis emphasize that changes in gut microbiota composition may play an important role in the development of Alzheimer’s disease [126,127]. These findings are supported by the discovery that individuals with amyloid deposition exhibit a higher abundance of pro-inflammatory bacteria and a lower abundance of anti-inflammatory bacteria compared to those without amyloid deposition within the brain [128].

Therefore, microbiota may interfere with the pathophysiology of AD through various mechanisms (see Table 3). For instance, gut dysbiosis may play a role in the development of Alzheimer’s disease by facilitating the aggregation of Aβ proteins and inciting neuroinflammation through a variety of intermediary substances. Thus, several bacterial species that inhabit the gut have been demonstrated to have the ability to generate functional amyloids, including curli and CsgA (*Escherichia coli*, *Salmonella enterica*, *Citrobacter koseri*), Fap (*Pseudomonas fluorescens*), TasA (*Bacillus cereus*), Bap, and PSM (*Staphylococcus* spp.) [129,130]. These bacteria-derived amyloids serve as instigating factors for cross-seeding interactions involving bacterial and human amyloidogenic proteins, enabling the development of misfolded Aβ oligomers and fibrils [131,132]. Moreover, another mechanism involved in the association of dysbiosis with Alzheimer’s disease is the potential of microbiota impairments to facilitate the synthesis of amyloid-beta, a process that can cause neurocognitive deficits [133]. These deficits have been correlated with significantly reduced BDNF concentrations in the mature brain [134].

The dysfunction of the intestinal microbiota also leads to neuroinflammation and neuronal apoptosis by stimulating the release of lipopolysaccharide (LPS) and other endotoxins from Gram-negative bacteria [135]. However, bacterial exotoxins are also linked to AD pathogenesis by affecting the integrity of the intestinal mucosal barrier. Consequently, an elevated prevalence of pathogenic bacteria such as *H. pylori*, *Salmonella* spp., and *E. coli* in the gut microbiota can lead to bacterial translocation and trigger an immune response, ultimately inducing the accumulation of certain inflammatory mediators that can penetrate the brain, especially as the blood–brain barrier becomes more permeable with age [127,136]. Other studies suggest that a decrease in gut flora can contribute to the pathogenesis of AD by affecting the development and function of microglia, resulting in the atypical clearance of Aβ and tau protein [137]. Furthermore, intestinal dysbiosis has been shown to lead to the activation of pro-inflammatory microglia through the accumulation of various metabolites, such as phenylalanine and isoleucine, which in turn induce the differentiation and proliferation of pro-inflammatory T helper 1 (Th1) cells [138].

**Table 3:** The role of microbiota in AD pathophysiology.

Mechanisms of the Microbiota in AD	References
Functional amyloids generated by several bacterial species	[129,130]
Facilitating the synthesis of amyloid-beta (Aβ) → neurocognitive deficits	[133]
Bacterial endotoxins → neuroinflammation and neuronal apoptosis	[135]
Bacterial exotoxins → impaired integrity of the intestinal mucosal barrier	[127,136]
Activation of pro-inflammatory microglia	[138]
Atypical clearance of Aβ and tau protein	[137]
Short-chain fatty acids (SCFAs), the main metabolites resulting from gut microbial fermentation	[139]
Reducing the concentration of N-Methyl-D-aspartate receptors (NMDA) within the hippocampus	[140]

Recently, it has been demonstrated that short-chain fatty acids (SCFAs) arising from the gut microbial metabolism may play pivotal roles in the pathogenesis of Alzheimer’s disease due to their diverse effects, both advantageous and detrimental. Consequently, they have the capacity to regulate brain metabolism, along with the expression of genes associated with Alzheimer’s disease, including H4K12, H4K5, and H3K14, while also maintaining the integrity of the blood–brain barrier. Moreover, short-chain fatty acids have emerged as a prominent topic of investigation regarding their potential to inhibit neuroinflammation and disrupt amyloid protein formation [139].

The correlation between the administration of broad-spectrum antibiotics and the emergence of Alzheimer’s disease has also been strengthened, based on their potential to disrupt the balance of the gut microbiota [133]. For example, the administration of ampicillin to postweaning male rats has been shown to lead to Alzheimer’s disease by reducing the concentration of N-Methyl-D-aspartate receptors (NMDA) within the hippocampus, which is accompanied by deficits in spatial memory [140].

Targeting the gut microbiota in AD may seem to be an innovative therapeutic approach. Therefore, recent findings have highlighted a new algae-derived therapeutic agent, named GV-971, which can mitigate neuroinflammation by altering the structure of the gut microbiome, leading to a reduction in the peripheral levels of phenylalanine and isoleucine. This, in turn, ultimately culminates in substantial cognitive improvement [138]. Other studies have demonstrated the anti-Alzheimer’s role of probiotic administration, in both human and animal studies. Thus, it may improve synaptic plasticity and spatial memory and reduce amyloid plaques within the hippocampus of treated mice. Meanwhile, human studies have demonstrated improvements in metabolic profiles, cognitive abilities, and memory capacities in individuals with AD through probiotic supplementation. Additionally, probiotic exopolysaccharides may also exert beneficial effects, such as reducing neuroinflammation and neuronal apoptosis, inactivating acetylcholinesterase and tyrosinase, in addition to their antioxidant properties [126].

Moreover, several vaccines are currently undergoing phase 2 studies as a potential therapeutic option for AD. Therefore, Bacillus Calmette-Guerin (BCG), initially employed against Mycobacterium tuberculosis, has been shown to increase circulating IFN γ levels, enhance anti-inflammatory cytokines within the brain, and recruit macrophages to cerebral Aβ plaques. This ultimately leads to the restoration of cognitive decline in a transgenic mouse model of Alzheimer’s disease [141]. Furthermore, the Tdap vaccine, which typically guards against tetanus, diphtheria, and whooping cough, has been linked with a 42% decrease in the risk of dementia. This is supported by evidence suggesting that *Bordetella pertussis*, which causes whooping cough, could play a role in neuroinflammation and neurodegeneration, as well as in the formation of tau tangles and Aβ plaques in Alzheimer’s disease [142].

## 11. The Infectious Hypothesis of Alzheimer’s Disease

Numerous studies have begun to explore the role of infections in the development of Alzheimer’s disease (see Table 4) [143]. For example, the potential involvement of *Chlamydia pneumoniae* in the initial phases of AD pathogenesis has been improved. This is supported by the discovery that this bacterium may contribute to the development of amyloid deposits in infected mice. Furthermore, the presence of *C. pneumoniae* was detected in the brains of individuals with Alzheimer’s disease, indicating the susceptibility of neurons, astrocytes, and microglia to this bacterial infection [144,145]. Furthermore, studies in infected mice have shown that under acute stress conditions, *Citrobacter rodentium*, a Gramme-negative bacterium, can induce an increase in pro-inflammatory cytokine levels within the colon, a reduction in the expression of brain-derived neurotrophic factor (BDNF) in the hippocampus, and memory deficits [143]. Other studies have suggested that *Helicobacter pylori* (HP) may potentially contribute to the development of AD [146,147]. Therefore, the potential of this bacterium to induce tau protein hyperphosphorylation reminiscent of Alzheimer’s disease characteristics was demonstrated in both in vitro neuronal cell cultures and in vivo rat brain models by activating glycogen synthase kinase-3β (GSK-3β) [147]. As a result, HP eradication has been shown to increase longevity among AD patients [146]. These findings are further supported by the beneficial effects of minocycline administration in Alzheimer’s disease. Thus, this antibiotic has been demonstrated to reduce neuroinflammation in AD by diminishing the release of pro-inflammatory cytokines like IL-6 and TNFα. Additionally, it exhibits anti-tau and anti-Aβ effects, leading to improvements in cognitive function in rats [71].

Animal studies have shown that infection significantly accelerates the development of Aβ plaques in murine models of AD with brain infection. Furthermore, it is suggested that the Aβ peptide is involved in a comprehensive innate immune response, as evidenced by the presence of various pathogens, such as bacteria (*Borrelia burgdorferi*), viruses (Herpes Simplex virus type 1), fungi, and parasites in postmortem brain tissue from Alzheimer’s disease [148].

Moreover, recent studies indicate a correlation between Varicella-Zoster virus (VZV) infections and Alzheimer’s disease pathology. This association is indirectly supported by the influence of VZV on gliosis and the exaggerated release of inflammatory cytokines [149]. However, Herpes Simplex virus type 1 (HSV1) exhibits the most prominent association among herpes virus types with Alzheimer’s disease pathology, exerting direct and indirect effects that result in significant neuroinflammation. It has been detected in the autopsied brain tissue of patients with Alzheimer’s disease in various regions, including the cerebral cortex and the cerebellum, and has been demonstrated to trigger the deposition of Aβ plaques both intracellularly and extracellularly through various molecular pathways. Furthermore, HSV-1 has been correlated with the elevated hyperphosphorylation of tau protein in individuals with Alzheimer’s disease at specific serine threonine-proline positions, potentially improving the activity of cyclin-dependent kinase 5, protein kinase A, and glycogen synthase kinase [150]. Consequently, valacyclovir, recognized for its inhibition of HSV replication, is also undergoing clinical trials for Alzheimer’s disease, considering the evidence suggesting the involvement of HSV in AD [142].

**Table 4:** The interference of bacterial and viral infections in the occurrence of AD.

Type of Infection	Infectious Agent	Mechanisms in Alzheimer’s Disease	References
Bacterial	*Chlamydia pneumoniae*	Development of amyloid deposits in infected mice	[144,145]
Bacterial	*Citrobacter rodentium*	Under acute stress conditions: - Increasing the levels of pro-inflammatory cytokines within the colon - Reduction in the expression of brain-derived neurotrophic factor (BDNF) in the hippocampus	[143]
Bacterial	*Helicobacter pylori*	Inducing tau protein hyperphosphorylation by activating glycogen synthase kinase-3β (GSK-3β)	[147]
Bacterial	*Bordetella pertussis*	- Formation of tau tangles and Aβ plaques - Inducing neuroinflammation and neurodegeneration	[142]
Viral	VZV	- Exaggerated release of inflammatory cytokines - Facilitating gliosis	[149]
Viral	HSV1	- Inducing tau hyperphosphorylation at specific serine threonine–proline positions by improving the activity of cyclin-dependent kinase 5, protein kinase A, and glycogen synthase kinase (GSK) - Triggering the deposition of Aβ plaques intracellularly and extracellularly - Leading to significant neuroinflammation	[150]

## 12. Vascular Hypothesis

There is growing evidence that vascular dysfunction plays a significant role in the progression of AD [151]. Thus, neuropathological studies have indicated that 92% of patients with Alzheimer’s disease exhibit cerebral arteriosclerotic changes [152]. According to the “vascular hypothesis”, it is considered that the dysregulation of cerebral blood flow (CBF), along with blood–brain barrier impairment, may be responsible for the pathogenesis of AD. These cerebrovascular dysfunctions can lead to synaptic impairment and neuronal degeneration by releasing various neurotoxic substances within the brain and also by decreasing Aβ clearance [151,153].

Vascular disturbances in AD may result from the down-regulated expression of both the mesenchyme homeobox 2 (MEOX2), a homeodomain transcription factor associated with angiogenesis and smooth muscle cell migration, and the lipoprotein receptor-related protein (LRP), a crucial molecule for Aβ clearance, in endothelial cells. The importance of MEOX2 is supported not only by the finding that its absence in mice leads to a 50% reduction in cortical cerebral blood flow, but also by the fact that the restoration of MEOX2 expression may contribute to increased angiogenesis in AD brains [154].

Animal postmortem studies have also highlighted the correlation between vascular smooth muscle cell (VSMC) dysfunction and a neuroinflammatory phenotype, characterized by the production of several inflammatory mediators, including monocyte chemoattractant protein (MCP)-1, MMP-9, and CD68, along with the presence of hyperphosphorylated tau protein [155]. Other animal studies have revealed that the introduction of APOE-ε4 in 5xFAD Alzheimer’s disease mice is linked to BBB impairment and decreased CBF, mediated by the up-regulation of cyclophilin-A-metalloproteinase-9 signaling within pericytes. Consequently, the inhibition of this pathway may have beneficial effects in the mouse model of AD, reducing neuronal apoptosis, alleviating BBB impairment, and ultimately resulting in improved cognitive performance [156].

Furthermore, numerous studies have underscored the therapeutic promise of antihypertensive medications in Alzheimer’s disease, given their capacity to mitigate the onset of cognitive decline [157]. Therefore, telmisartan and perindopril have been shown to improve brain atrophy, as indicated by ventricular enlargement after 12 months of treatment [142].

## 13. Autophagy

Autophagy, predominantly initiated by the stimulation of the AMP-activated protein kinase (AMPK) pathway and the suppression of the nutrient starvation-mediated mammalian target of the rapamycin complex 1 (mTORC1) pathway, is a fundamental cellular process. This activation and inhibition cascade triggers the formation of a double-membrane autophagosome, which involves numerous proteins, such as the Unc-51-like autophagy activating kinase (ULK1/2), vacuolar protein sorting 34 (VPS34), and Beclin1. During this phase, damaged organelles and proteins are sequestered in the autophagosome through interactions with Atg8/microtubule-associated proteins 1A/1B light chain 3B (LC3) and adaptor proteins such as sequestosome-1 (p62/SQSTM1). Subsequently, the autophagosome merges with a lysosome, forming an autolysosome, where the engulfed cargos are degraded by lysosomal hydrolases [158].

Dysfunction in the autophagy process has been increasingly recognized as a contributing factor to the development of Alzheimer’s disease [159,160,161]. Consequently, in the brains of patients with AD, an accumulation of autophagosomes and autolysosomes is observed, coupled with the down-regulation of PI3P, mediated by the VPS34 complex. Furthermore, key components of the VPS34 complex, particularly Beclin-1, have been found to undergo a substantial reduction as the disease progresses [161]. Autophagy plays a crucial role in both tau pathophysiology and in Aβ metabolism, thus inhibiting autophagic flux led to impaired tau clearance, resulting in the extensive accumulation of insoluble tau aggregates [9].

The particular autophagic mechanism targeting impaired mitochondria is called mitophagy and has been observed to be affected in postmortem investigations of Alzheimer’s disease, showing a significant reduction in the hippocampal region. Furthermore, the finding of compromised mitophagy in the entorhinal cortex (EC), which is the region primarily affected at the beginning of the disease, may serve as an early hallmark of AD [161].

Contemporary research has shown that the enhancement of autophagy-related proteins and pathways may offer therapeutic benefits. Therefore, in the context of Alzheimer’s disease research, particularly in the mouse model APP/PS1, the inhibition of the mammalian target of rapamycin (mTOR) signaling through the PTEN/PI3K/Akt pathway has been identified as a facilitator of the autophagic degradation of amyloid-beta, also associated with improved cognitive function [159,160]. Consequently, several compounds, such as rapamycin, memantine, nilotinib, and even natural products such as curcumin, have been shown to induce autophagy through the inhibition of mTOR. Moreover, a multitude of molecules have been found to enhance the autophagy process in AD through the activation of the AMPK pathway (e.g., lithium and glucosamine), while others may serve as AMPK activators and mTOR inhibitors (e.g., metformin and oleuropein) [161]. Furthermore, recent research has identified latrepirdine as a significant stimulator of autophagy through an mTORC1 and Atg5-dependent mechanism and resveratrol as a versatile compound that acts as an activator of AMPK and sirtuin-1 [161,162].

In the context of targeting autophagy stimulation within microglia as a novel therapeutic approach, several molecular pathways have been demonstrated to facilitate Aβ clearance. These include the STK11/LKB1 (serine/threonine kinase 11)-mediated AMPK pathway, p21-activated kinase 1 (Pak1), and the Axl tyrosine kinase receptor [163,164]. In addition, triggering the transcription factor EB (TFEB), a key regulator of lysosomal biogenesis, could enhance the autophagy-lysosomal pathway within astrocytes, resulting in the increased uptake and degradation of Aβ, thus also mitigating tauopathy. As a result, trehalose has recently been investigated as a TFEB activator in Alzheimer’s disease [161,165]. Other drugs are currently being studied as potential therapies for AD, facilitating autophagy through various mechanisms. These include the inhibition of myo-inositol-1-phosphate synthase (e.g., carbamazepine, valproate), promotion of VPS34 complex generation and modulation of Beclin-1 (e.g., spermidine), activation of mitophagy (e.g., nicotinamide riboside), reduction in calpain activation (e.g., calpeptin, calpastatin), and modulation of the Ca^2+^-calpain Gsα pathway via βCa^2+^ channel blockers like nilvadipine [161,162].

Apart from pharmacological strategies, gene therapy is increasingly being explored as a novel approach to stimulate autophagy in AD, utilizing various techniques such as the AAV/Aβ vaccine, microRNA methods, and also enhancing Beclin-1 expression in mouse models through lentivirus encoding. All of these have been shown to significantly promote the clearance of tau and Aβ in the brains of AD mice [162].

## 14. The Glymphatic and Meningeal Lymphatic Systems

Prominent risk factors associated with Alzheimer’s disease, such as advanced age [166], neurovascular damage [167], and sleep disturbances [168], have been identified as having a correlative relationship with the decreased functionality of both the glymphatic and meningeal lymphatic systems [169,170,171,172]. Furthermore, dysfunctions within the glymphatic system have been identified as a contributing factor to the onset of neuroinflammation, which in turn amplifies the progression of Alzheimer’s disease [173]. The glymphatic system represents a comprehensive perivascular network within the brain, which serves a vital role in facilitating the recirculation of cerebrospinal fluid (CSF) throughout the brain parenchyma. This system is essential to support the elimination of interstitial solutes, including amyloid-beta and tau protein [174].

Aquaporins represent a distinct family of transmembrane proteins that serve as selective water channels [175]. Among these, aquaporin-4 (AQP4) is the most prevalent water channel within the brain, characterized by its primary location around blood vessels in the plasma membranes of astrocytes [176]. Aquaporin-4, which is postulated to serve as the central regulator of the glymphatic system clearance mechanism, is known to be ubiquitously expressed among astrocytes [9,177]. A predominant concentration of AQP4 is located at the ends of the feet of astrocytes, a configuration described as the polarized distribution of AQP4. This specific location facilitates the connection between the astrocyte cytoplasm and the interstitial fluid (ISF), allowing for the effective movement of the interstitial fluid [177,178].

Numerous investigations have established a correlation between the aquaporin-4 depolarization and the pathological manifestations of Alzheimer’s disease. This association has been noted in human postmortem studies of frontal cortex tissues, suggesting a significant link between altered AQP4 polarization and AD neuropathology [174,179,180]. The alteration in the polarization of aquaporin-4 has been hypothesized to impact the performance of the glymphatic system in the removal of waste products. This change in AQP4 orientation is believed to lead to decreased cerebrospinal fluid flow and the subsequent accumulation of metabolic by-products [91,181]. Recent research has indicated that the disruption of the location of perivascular aquaporin-4 in neurodegenerative diseases, such as Alzheimer’s disease, could contribute to the increased vulnerability of the aging brain to protein misaggregation [174].

AQP4 is attached to the end of the feet of astrocytes via the dystrophin-associated complex (DAC), which encompasses alpha-syntrophin (SNTA1) and dystroglycan (DAG1). Recent research has indicated a correlation between elevated levels of alpha-syntrophin (SNTA1) and dystroglycan (DAG1) with an increase in the accumulation of tau proteins in the temporal cortex [182]. There is a scientific hypothesis suggesting that the MLC1 gene, known for its association with elevated amounts of tau protein, encodes an astroglial membrane transporter. This transporter is believed to be functionally connected to AQP4 and the dystrophin-associated complex, indicating a complex interaction between these molecular components in the astrocytic membrane [183].

Recent research has revealed that the elimination of AQP4 in the cerebral tissues of transgenic mice engineered to express the P301S tau mutation leads to a notable increase in the formation of tau proteins within the cerebrospinal fluid. This process significantly exacerbates tau protein aggregation, ultimately leading to pronounced neurodegenerative effects [184]. Polymorphisms in the aquaporin-4 gene have been identified as being correlated with Aβ accumulation, advancement in disease stages, and decreased cognitive abilities. These genetic variations are likely indicative of modifications in the functionality of the glymphatic system, specifically its capacity to clear Aβ from the brain [185].

Sleep has been scientifically recognized as a crucial determinant of the efficacy of the glymphatic system. This is characterized by an increase in the CSF flow during sleep, which in turn facilitates the better clearance of interstitial waste, including soluble amyloid-beta [172]. Research conducted in both human subjects and animal models has consistently shown an increase in Aβ levels after periods of sleep deprivation [186,187]. Additionally, recent research has indicated that certain genetic polymorphisms in the aquaporin-4 gene may exert a direct impact on sleep quality [182], while other studies have shown that mice subjected to sleep deprivation exhibit a depolarization of AQP4 [188], suggesting a reciprocal relationship.

Regarding age as the predominant risk factor for neuropathologies associated with protein aggregation [189], in aged murine models, a pronounced decline in the cerebrospinal fluid–interstitial fluid exchange has been documented, with a notable 40% reduction in the clearance of Aβ injected into the brain parenchyma [171]. This decrease has been associated with a reduction in CSF production and decreased arterial pulsatility, factors that could impact glymphatic influx and contribute to the pathogenesis of Alzheimer’s disease [171,190]. Furthermore, these observations were accompanied by a loss of perivascular aquaporin-4, suggesting that AQP4 polarization is compromised in the older brain [171].

In mouse models of Alzheimer’s disease, altered meningeal lymphatic vessels (MLVs) were found to exacerbate the pathology of AD, as evidenced by increased amyloid-beta aggregation in the meninges and a higher burden of Aβ plaques in the hippocampus. However, this process appears to operate independently of astrocytic aquaporin-4, as both the location and the amount of AQP4 were found to remain unchanged, indicating a distinct mechanism of action in the progression of AD pathology [10]. In the context of aging, there is a noted association with changes in the meningeal lymphatic system, particularly characterized by immunosenescence. This is evidenced by the decreased expression of the CCR7 receptor and a concurrent increase in the accumulation of T cells within the meningeal tissue in older mice. In particular, the genetic deletion of CCR7 results in a considerable decrease in polarized aquaporin-4 expression and leads to a compromised glymphatic influx of cerebrospinal fluid in adult mice [191]. In addition, the elimination of the CCR7 gene has been found to exacerbate AD-related pathologies in a familial murine model of AD. This includes an increase in amyloid-beta deposition, enhanced cerebral vascular damage, and increased microglial activation. Additionally, this knockout leads to a deterioration of the cognitive profile, underscoring the critical role of CCR7 in modulating the progression and severity of AD-related pathological changes [191,192].

Within the context of AD treatment, targeting aquaporin-4 is emerging as a particularly promising therapeutic strategy. The efficacy of AQP4-centric drugs has been established in the regulation of several critical neurological processes. These include the modulation of the Ca^2+^ signaling pathway, maintenance of K+ balance, facilitation of glutamate transport, and regulation of astrocyte proliferation, activation, and neuroinflammatory responses [176]. Additionally, nonpharmacological interventions such as physical exercise, noninvasive brain stimulation, traditional Chinese medicine, and nutritional supplementation (like polyunsaturated fatty acids) have been shown to result in the modulation of polarized aquaporin-4 expression, increased CSF glymphatic flow, the improved clearance of interstitial fluid solutes, and also in the morphology of the meningeal lymphatics. In animal models of Alzheimer’s disease, these approaches have been correlated with a reduction in amyloid-beta deposition, a decrease in reactive gliosis, and a decrease in neuronal loss, which ultimately results in increased cognitive abilities, specifically in the domains of learning and memory [192].

## 15. Metals in Alzheimer’s Disease

The involvement of metals in the development of AD remains a subject of ongoing debate, with some studies postulating a link between initial exposure to heavy metals and the subsequent emergence of AD. Moreover, therapeutic strategies grounded in the metal hypothesis of AD have evolved along two distinct trajectories. An approach involves the administration of metal supplements. For example, numerous clinical investigations have examined the impact of zinc (Zn) and copper (Cu) supplementation on cognitive performance. On the contrary, there exists a perspective that emphasizes the therapeutic potential of chelating excess zinc, copper, or iron (Fe) metals based on their ability to catalyze amyloid-beta aggregation [193].

### 15.1. Heavy Metals

#### 15.1.1. Arsenic (As)

Arsenic is ingested primarily through contaminated water, although it can also be found in soil and air [193]. Epidemiological research indicates a correlation between arsenic exposure and cognitive decline [194], with elevated levels of arsenic in soil associated with an increased mortality rate related to Alzheimer’s disease [195]. Furthermore, in animal studies, exposure to arsenic has been linked to the onset of memory impairment [193]. Exposure to arsenic has also been found to induce tau aggregation and hyperphosphorylation [193], elevate amyloid-beta levels [196], and cause oxidative stress predominantly through mitochondrial dysfunction [197]. Additionally, this exposure is associated with neuroinflammation, vascular damage, neuronal apoptosis and necrosis [193].

#### 15.1.2. Lead (Pb)

Lead constitutes an environmental contaminant that can be encountered through water, food, and air. Empirical research has established a link between exposure to lead and pathological alterations associated with AD. Specifically, lead has been demonstrated to interact with amyloid-beta, enhance its production and aggregation, and augment tau hyperphosphorylation. Furthermore, lead triggers oxidative stress, compromises the blood–brain barrier, disrupts intracellular cation homeostasis by interfering with calcium homeostasis and substituting zinc ions in zinc-dependent enzymes, and also induces epigenetic modifications by altering the expression of genes related to Alzheimer’s disease [193,198]. Prolonged exposure to lead has been found to precipitate neurodegenerative processes in rat models, primarily through mechanisms that involve demyelination, apoptosis, and oxidative stress. This results in compromised motor functions and distinct neurodegenerative characteristics [199]. In senescent mice, exposure to Pb during adolescence has been associated with disrupted autophagy in the brain, characterized by molecular alterations such as decreased p-mTOR/mTOR ratios, elevated LC3II/LC3I ratios, the increased expression of Atg7, Atg12 and Beclin-1, and up-regulated p62 [200].

Regarding the impact of lead on oligodendrocytes, it has been shown that lead induces structural abnormalities in the cerebellum and malformations in the myelin sheath by decreasing the population of oligodendrocytes and glycoproteins associated with myelin [198]. Exposure to lead also instigates neuroinflammatory responses by inducing microglial activation, facilitating caspase-1 cleavage, improving NLRP3 expression, increasing autophagic protein levels, and promoting NF-kB phosphorylation [201]. Furthermore, Pb induces detrimental impacts on microglial cells by decreasing glutathione levels and amplifying the expression of Nrf2, consequent to increased oxidative stress [198]. Similarly to As and Cd, the exposure of astrocytes to Pb has been observed to induce apoptosis by increasing levels of peroxisome proliferator activated receptor gamma (PPARγ) and facilitating its interaction with the poly (ADP-ribose) polymerase gene (PARP) and PPARγ-response elements (PPREs) [202].

#### 15.1.3. Copper (Cu)

In Alzheimer’s disease, disrupted copper homeostasis contributes to disease progression by promoting the formation of toxic amyloid-beta oligomers and interacting with amyloid precursor protein (APP). Copper accumulation in amyloid plaques, along with its role in tau protein phosphorylation and aggregation, further exacerbates the pathology of AD. Moreover, the differential binding affinity of copper with apolipoprotein E (ApoE) isoforms, particularly the higher affinity of ApoE2 and the lower affinity of ApoE4 for copper, plays an important role in the development of the disease [193]. Copper also disrupts neuroprotective pathways, instigates oxidative damage, and facilitates apoptosis by suppressing the p-CREB/ BDNF pathway, modifying the Bcl2/Bax ratio and diminishing mitochondrial membrane potential. Furthermore, copper precipitates neuroinflammation and neuronal death by enhancing DNA fragmentation and amplifying the expression of p53-mediated apoptotic proteins. Exposure to copper results in the reduced expression of Parkin and PINK1, an altered LC3BII/I ratio, the up-regulation of proteins within the NLRP3/caspase1/GSDMD axis, a decrease in mitochondrial membrane potential, elevated levels of reactive oxygen species (ROS), and the increased expression of P62 [198]. Moreover, copper has been shown to facilitate glutamate toxicity in astrocytes, affect memory and learning abilities, and contribute to higher rates of astrocytosis and apoptosis [203]. Furthermore, it amplifies the expression of genes associated with neurodegeneration and destabilizes microglial equilibrium, thus intensifying cognitive deterioration [204].

#### 15.1.4. Cobalt (Co)

Cobalt is a fundamental component of vitamin B12 and is recognized simultaneously as an environmental toxin [193]. It instigates neurotoxic effects by modulating the activity of trans and cis phosphorylated tau proteins through the Pin1-mediated regulatory pathway [198]. Moreover, mice exposed to cobalt have been shown to exhibit neurodegeneration correlated with aging [193].

#### 15.1.5. Cadmium (Cd)

Exposure to cadmium in humans, mainly from food, air, and water, leads to significantly higher levels of cadmium in smokers compared to non-smokers. Cadmium may play a role in the pathogenesis of AD. Thus, human studies have established an association between cadmium exposure and elevated mortality related to AD, as well as cognitive decline [193]. Ruczaj and Brzoska have suggested that the primary mechanism of cadmium’s effects is the induction of oxidative stress [205]. However, cadmium also interacts with amyloid-beta, enhancing its aggregation and promoting tau hyperphosphorylation and aggregation. It alters the blood–brain barrier, disrupts cholinergic transmission, and leads to the death of cholinergic neurons in the basal forebrain [193]. Additionally, cadmium disrupts intracellular cation homeostasis by acting as an antimetabolite of zinc, replacing it in zinc-dependent enzymes [206].

Regarding the impact of cadmium on neuronal function, exposure to cadmium has been described as contributing to the induction of apoptosis, cognitive decline, and neuroinflammation. This is mainly attributed to increased lipid peroxidation and oxidative stress, together with the dysregulation of the Nrf-2 and NF-κB pathways [198]. Cadmium is also capable of inducing apoptosis and neurodegeneration by promoting the accumulation of autophagosomes. This accumulation is the result of impaired autophagic flux in neuronal cells, which is a consequence of the dysfunction of the Akt signaling pathway [207]. Furthermore, it activates the JNK pathway, leading to the up-regulation of autophagy-related (Atg) protein expression, expansion of autophagosomes, and subsequent induction of apoptosis [208]. Regarding the impact on microglial function, cadmium has been shown to promote microglial proliferation and activation by activating the Wnt/β-catenin signaling pathway. Additionally, it contributes to neuroinflammation by modulating the expression levels of ionized calcium-binding adapter molecule 1 (IBA-1), glial fibrillary acid protein (GFAP), and a variety of inflammatory mediators [198]. The exposure of astrocytes to cadmium leads to the fragmentation of intracellular DNA, along with the suppression of astrocytic proliferation. Furthermore, it facilitates the induction of apoptosis through the up-regulation of Bax and the down-regulation of Bcl2 and promotes the decreased expression of mRNAs from antioxidant enzymes [209].

#### 15.1.6. Mercury (Hg)

Exposure to mercury occurs through food ingestion, the inhalation of air, and the consumption of water, with seafood consumption being identified as the predominant contributor to mercury poisoning. Although there are some controversies regarding the contribution of mercury in the development of Alzheimer’s disease, a variety of molecular mechanisms have been described, including the hyperphosphorylation and aggregation of tau protein, the promotion of amyloid-beta production and aggregation, the disruption of calcium homeostasis, along with the induction of oxidative stress [193].

#### 15.1.7. Zinc (Zn)

Contemporary studies indicate that zinc plays a role in the promotion of neurodegeneration [198]. Thus, it is involved in the promotion of tau protein phosphorylation, aggregation, and translation. Furthermore, Zn exhibits accumulation within amyloid plaques, interacts with amyloid-beta, and fosters Aβ aggregation and plaque formation [193]. It also triggers necroptosis in astrocytes, leading to subsequent inflammation and altered neurobehavioral functions [198]. Zinc has been shown to decrease neurobehavioral functions by intensifying oxidative stress, promoting microglial activation, initiating cytochrome c release, increasing pro-inflammatory cytokine production, facilitating Bax translocation to mitochondria, and by activating caspase 3/9 [210]. Studies have shown mixed results on the impact of zinc supplementation in Alzheimer’s disease. Consequently, the incorporation of zinc into the diet has been proposed to improve cognitive functions in AD individuals, while contrasting findings indicate that zinc supplementation in AD does not have a significant advantage [193].

### 15.2. Other Metals in AD

#### 15.2.1. Aluminum (Al)

Exposure to aluminum has been shown to be correlated with cognitive and behavioral impairments, neuronal damage, and the disturbance of the blood–brain barrier. The encounter with aluminum induces pyroptosis by triggering the assembly of the NLRP3 inflammasome, activating CASP1, and facilitating the release of IL-1β and IL-18, which in turn stimulate microglial activation [211]. In brain areas affected by AD, including the amygdala, the hippocampal region, and the entorhinal cortex, elevated concentrations of Al have been documented. The simultaneous deposition of aluminum and fibrillar amyloid-beta within amyloid plaques has been detected in brain tissue samples from individuals with familial Alzheimer’s disease (fAD), particularly those carrying the PSEN1-E280A (Glu280Ala) mutation, a variant linked to a more severe form of AD [193]. Regarding the impact of Al exposure on astrocytes, studies have shown that aluminum treatment leads to an elevation in reactive oxygen species (ROS) levels, a decrease in the activities of antioxidant enzymes, specifically catalase and superoxide dismutase, and culminates in cell death within astrocytes [198].

#### 15.2.2. Manganese (Mn)

Elevated concentrations of manganese have been associated with reduced cognitive performance, and an increase in Mn levels has also been observed in individuals diagnosed with AD [193]. Exposure to Mn during developmental stages is associated with alterations in NF-kB signaling, leading to neuroinflammation characterized by an increased presence of neurotoxic astrocytes expressing C3 [198]. Furthermore, exposure to manganese precipitates mitochondrial dysfunction in astrocytes, characterized by the increased generation of reactive oxygen species (ROS), compromised respiratory functions, and the inhibited autophagic lysosomal degradation of impaired mitochondria, mediated by (TFEB) [212]. Regarding the impact of manganese on microglia, it has been demonstrated that Mn leads to neuroinflammation through a variety of mechanisms. Therefore, Mn intensifies neuroinflammation by disrupting the SIRT1/STAT3/PGC-1α signaling pathway, leading to an imbalance in microglial activation, with a shift toward a pro-inflammatory state [213]. Concurrently, manganese facilitates neuroinflammation by enhancing the expression and functionality of the PSMB8 immunoproteasome subunit in microglia [214]. It may also suppress the expression of p53 in microglia by DNA methylation, subsequently leading to the up-regulation and hyperexpression of cyclooxygenase-2 (COX-2), a crucial mediator in neuroinflammation [215]. Furthermore, Mn stimulates the production of neuroinflammatory cytokines by elevating NF-kB (p65) mRNA levels and facilitating the phosphorylation of p65 in microglia. Concurrently, it triggers the release of inflammatory cytokines via the JAK2/STAT3 signaling pathway, culminating in neuronal apoptosis [198].

#### 15.2.3. Magnesium (Mg)

Research involving human subjects has elucidated that a deficiency in magnesium is associated with memory impairment, while magnesium supplementation can ameliorate memory functions in patients with dementia. Magnesium affects the processing and transport of amyloid precursor protein, with decreased Mg concentrations promoting the beta-secretase pathway and elevated Mg amounts favoring the alpha-secretase pathway. Furthermore, the administration of magnesium sulfate in experimental animals has been found to mitigate tau phosphorylation and exert an influence on the preservation of synaptic plasticity and cognitive functions [193].

### 15.3. Calcium (Ca^2+^) Hypothesis

The dysregulation of calcium ions represents a frequent pathophysiological occurrence in the context of Alzheimer’s disease, constituting one of the initial phenomena in the onset of this disorder [216,217]. Zhong et al. [218] have shown that the N-methyl-D-aspartate receptor subunit (GluN3A) is crucial to maintaining consistent Ca^2+^ homeostasis, and its deficit contributes to the pathogenesis of AD. Their investigation of molecular, cellular, and functional alterations in adult/senescent GluN3A knockout mice led to the inference that chronic “degenerative excitotoxicity” could induce sporadic AD. They identified GluN3A as a primary pathological element, proposing that a moderate but continuous Ca^2+^ overload throughout life is a causative pathogenic mechanism in sporadic AD. Consequently, GluN3A emerges as a potential therapeutic target independent of amyloid pathways.

Although the specifics of cytosolic calcium changes in Alzheimer’s disease remain incompletely characterized, various calcium-permeable channels, pumps, receptors, and transporters have been implicated at both neuronal and glial levels. Additional pathophysiological mechanisms contributing to calcium dyshomeostasis include the activation of transient receptor potential channels, voltage-dependent calcium channels of type L, and ryanodine receptors, among others [12].

Calmodulin, a predominant regulatory protein, plays a vital role throughout the various stages of AD, significantly influenced by the presence of amyloid-beta. The interaction between amyloid-beta extends not only to calmodulin, but also to several calmodulin-binding proteins implicated in Alzheimer’s pathology [217]. Numerous pharmaceutical agents targeting calmodulin (CaM) and specific calmodulin-binding proteins (CaMBPs) are currently available. For instance, CaMKII and PP2B, which are two classic calmodulin-binding proteins (CaMBPs) essential in Alzheimer’s disease pathogenesis, represent prime models of comprehensive research. A substantial range of drugs have been formulated to inhibit CaMKII functionality, with a considerable number of additional compounds currently under development [219].

### 15.4. Ferroptosis

In recent years, a novel form of cell death, termed ferroptosis, has been identified [220]. Ferroptosis is an iron-regulated programmed cell death mechanism that has been observed in clinical samples of Alzheimer’s disease, which has been delineated in the recent scientific literature as a separate entity from apoptosis, necrosis, and autophagy [220,221]. Over the past decade, extensive research has elucidated various signaling pathways implicated in the regulation of ferroptosis in Alzheimer’s disease, such as iron metabolism pathways, redox homeostasis mechanisms, lipid metabolism processes, and several other potential regulatory factors [221]. Moreover, ferroportin (FPN) is known to be a critical iron-regulatory protein directly involved in the modulation of ferroptosis in Alzheimer’s disease. Therefore, a deficiency of FPN in the cortical regions results in elevated iron concentrations, causing hippocampal atrophy and memory impairments reminiscent of AD. On the contrary, the reinstatement of FPN expression has been observed to ameliorate memory deficits and mitigate ferroptosis in murine models of Alzheimer’s disease [222].

Recent research indicates that brain cells derived from patients with Alzheimer’s disease display characteristics similar to ferroptosis, both biochemically and morphologically. These include the inactivation of glutathione peroxidase 4 (GPX4), the degradation of glutathione, an imbalance in iron metabolism resulting in increased reactive oxygen species, mitochondrial irregularities, lipid peroxidation, and a specific set of regulatory genes associated with mitochondrial and lysosomal functionalities, such as SEPHS2, GPX4, SEPSECS, PSTK, along with components of the mTORC1 pathway, PSAP, ASCL4, and NQO1 [220,223,224]. Regarding lipid peroxidation, the examination of metabolic aberrations in postmortem brain tissue from patients with Alzheimer’s disease has revealed increased levels of ferritin and the enhanced expression of cystine/glutamate transporters and light chain subunits involved in this process [221]. Furthermore, it has been observed that fatty acid synthase inhibitors impede ferroptosis-induced lipid peroxidation, improving cognitive functions in APP/PS1 transgenic mouse models [225]. Moreover, Ferroptosis Suppressor Protein 1 (FSP1) exerts an antiferroptotic effect through its role in scavenging the reduction in NAD(P)H in the GPX4-mediated pathway, which reduces coenzyme Q10, thus inhibiting lipid peroxidation [226].

Iron metabolism disruption has been shown to be intimately associated with amyloid-beta, senile plaques, and neurofibrillary tangles [224]. Thus, iron can exacerbate the aggregation of toxic amyloid-beta [220]. Furthermore, ferroptosis can precipitate the anomalous aggregation of tau proteins, facilitated by the activation of GSK-3β and the proteasome system. It may also directly induce neuronal oxidative damage, contributing to the pathogenesis of Alzheimer’s disease [220,221]. Ferroptosis may also be involved in the neuroinflammatory processes observed in Alzheimer’s disease. The production of damage-associated molecular patterns (DAMPs) during ferroptosis initiates glial cell activation through neuroimmune pathways. These activated glial cells, in turn, secrete a cascade of inflammatory mediators that contribute to neuronal degeneration [221].

Recent studies indicate the significant potential in the development of Alzheimer’s disease prevention strategies, particularly through agents that are effective in scavenging free radicals, chelating iron, and mimicking GPX4, which have shown efficacy in inhibiting ferroptosis [227].

## 16. Neuronal Cell Cycle Re-Entry in AD

Neuronal cell cycle re-entry has been identified as an early pathological event in AD, associated with hyperploidy and synaptic dysfunction, which may contribute to cognitive decline and increased neuronal death overtime [228,229]. Cultured cortical neurons manipulated to re-enter the cell cycle via SV40 large T antigen expression exhibited significant hyperploidy, the loss of axonal structure, and synaptic density reduction, which led to impaired synaptic activity and delayed cell death, paralleling changes observed in AD-affected neurons [228,230]. This process, once activated, shows two possible outcomes: an abortive re-entry leading to cell death or a non-abortive progression, where neurons complete DNA synthesis but ultimately die before the G2/M phase [229].

Studies suggest that cell cycle re-entry and hyperploidy may allow neurons to persist temporarily within functional networks but at the cost of gradual synaptic dysfunction, which could underpin early cognitive symptoms [229,231]. For instance, high potassium-induced membrane depolarization showed the partial restoration of synaptic activity and survival in neurons that re-entered the cell cycle, implicating a potential neuroprotective intervention [228]. Using mathematical models, researchers have demonstrated how feedback loops in the cell cycle and apoptotic signaling pathways sustain the pathological shift observed in AD, with transcriptional profiles similar to those found in AD brain tissues [232]. Targeting elements of these loops could provide disease-modifying effects, especially if combined with extracellular amyloid-beta (Aβ) reduction [232].

The overactivation of specific molecular pathways, such as ERK due to Aβ exposure, and the inactivation of cell cycle regulators such as APC/C-Cdh1 due to calcium dysregulation suggest a multifaceted role of cell cycle-related abnormalities in AD pathology [232,233]. 

The variability in cellular fate could be linked to the different stress levels that individual neurons encounter, such as oxidative stress or DNA damage [234].

Furthermore, neuronal re-entry into the cell cycle in the presence of oncogenes, such as c-myc and ras, has been shown to cause DNA replication, tau phosphorylation, and typical structural alterations of AD [235,236]. These studies introduce the concept that AD-like changes in neurons may arise from disrupted cell cycle control, with neurons often halting in the G2/M phase, exhibiting features of neurodegeneration observed in AD [235].

Evidence from AD models, including Drosophila and murine systems, supports a dual hypothesis in which cell cycle re-entry may predispose neurons to apoptosis or confer a protective effect by promoting polyploidy under certain conditions [234,237]. In mouse models, CCR was observed prior to Aβ plaque formation, suggesting that soluble Aβ oligomers (AβOs) are the key in triggering CCR, potentially mediated by insulin resistance pathways, which further implies therapeutic relevance for diabetes drugs in AD [238,239,240]. Experimental results indicate that drugs targeting AβO-related pathways and metabolic dysregulation, such as NMDA receptor antagonist memantine or GLP-1 agonists, could reduce CCR and delay neurodegeneration [240,241,242,243].

In contrast, most anticancer medications that restrict cell growth are likely to suppress neuronal CCR. Some FDA-approved cytotoxic chemotherapy drugs, with varying degrees of brain penetration, such as carmustine (strong) and paclitaxel (weak), have shown possible beneficial effects in preclinical models of AD [235].

These insights emphasize the importance of understanding neuronal cell cycle re-entry in AD and suggest that addressing this aberrant reactivation, alongside disrupting self-sustaining feedback loops, could yield effective strategies for modifying disease progression [232,242].

## 17. The Olfactory Pathway and AD

The accumulation of amyloid-beta (Aβ) peptides and tau protein, followed by the formation of neurofibrillary tangles and neuritic plaques in AD, may be responsible for the disruption of brain homeostasis, manifesting as cognitive deficits, particularly in memory [244]. During the early phases of Alzheimer’s disease, these pathological alterations are frequently observed in the olfactory bulb (OB), the entorhinal cortex (EC), and the hippocampus (HPC), all areas that are essential for memory and sensory processing [245]. A significant early indicator of AD is a reduction in olfactory function, which occurs before cognitive degeneration and serves as a distinctive biomarker for the early diagnosis of AD [246].

The anatomical structure of the olfactory system also makes it a vulnerable entry point for pathogens. Olfactory sensory neurons extend from the nasal cavity through the cribriform plate, creating a direct pathway to the OB, which has a comparatively weaker blood–brain barrier (BBB) [247]. This unique pathway may allow pathogens to bypass the BBB, enter the central nervous system (CNS), and trigger neuroinflammation [248]. Such breaches in the BBB facilitate pathogen infiltration into the CNS, potentially contributing to the early-stage inflammation observed in AD [249].

OB plays a pivotal role in the olfactory system, a pathway that links directly to regions of the brain related to memory. Positioned closely to the limbic areas, the OB coordinates memory-related processes, especially those that involve working memory [250]. The entorhinal cortex, meanwhile, acts as a critical gateway between the hippocampus and the medial prefrontal cortex (mPFC), facilitating rapid memory encoding [251]. This interconnected OB-EC-HPC-mPFC network underpins the encoding, processing, and retrieval of memories. As a result, any dysfunction in this circuit due to AD pathology, including plaque accumulation and cellular death, can significantly impair memory [252].

Epidemiological studies have underscored the link between olfactory function and cognitive health. For example, older adults with impaired olfaction face a substantially higher risk of developing cognitive decline [253]. Moreover, individuals carrying the APOE ε4 allele, a known genetic risk factor for AD, experience declines in odor sensitivity earlier than those without this allele [254]. Therefore, these studies could be the first step toward developing olfactory impairment as an early non-invasive biomarker of AD [255].

The role of the olfactory system in neurodegenerative diseases, particularly AD, has stimulated research into its therapeutic and diagnostic potentials. AD-related changes in OB could involve various mechanisms, including changes in receptor expression, neurogenesis, and degeneration [256]. A loss of olfactory function may precede cognitive symptoms in both AD and Parkinson’s disease, indicating the need for further research on the pathophysiological mechanisms underlying olfactory deficits in these conditions [257]. AD mouse models have mirrored these early olfactory impairments, which makes them valuable for studying neurodegeneration in the olfactory pathway and its potential impact on memory [258].

Regarding the potential therapeutic approaches of OB in AD, emerging evidence suggests that OB stimulation may be able to decrease Aβ plaque deposition and mitigate working memory deficits [259]. OB stimulation also enhances brain connectivity, particularly within the gamma frequency band, which is important for memory. In AD models, OB stimulation improved the connectivity in memory-relevant regions, with increased functional coherence between OB, EC, HPC, and mPFC. This connectivity helps prevent plaque accumulation and promotes neurogenesis within the OB-EC-HPC-mPFC network, providing further support for memory preservation [260].

Such findings suggest that OB stimulation can be effective as a preventive intervention, particularly for individuals with mild cognitive impairment (MCI), a condition that progresses to AD in up to 80% of cases in six years [259].

Given the accessibility of OB through the nasal cavity, OB stimulation could potentially be administered through minimally invasive nasal techniques. This pathway also opens possibilities for the development of novel therapeutic approaches targeting olfactory sensory neurons (OSNs) within the nasal cavity. By facilitating the early diagnosis and intervention of AD, such strategies could reduce the disease’s social and economic impact and potentially be applied to other neurodegenerative and neurocognitive disorders, including depression and epilepsy. The potential for minimally invasive stimulation methods targeting OB through the nasal cavity represents a transformative approach to AD and other CNS diseases [259].

## 18. Oral Health in AD

The association between poor oral health and an elevated risk of Alzheimer’s disease is receiving growing attention, with emerging evidence suggesting that declining oral health may contribute to the onset and progression of AD, particularly among older adults [261]. A large-scale analysis utilizing the TriNetX database classified more than 30 million anonymized individuals based on their oral health status, showing that the risk of developing AD is doubled for those with inadequate oral health in comparison to individuals who maintain good oral health. Among the dental issues analyzed, diseases linked to tooth loss were identified as the most significant risk factors for AD, underscoring an association between poor oral health and heightened AD risk [262].

Numerous studies also support a connection between declining oral health and AD, associating issues such as poor oral hygiene, tooth loss, cavities, reduced salivary flow, chronic periodontitis, mucosal infections such as Candida, and gum pain with a higher risk of cognitive decline, dementia, and AD [263,264,265,266,267,268,269]. Additionally, oral pathogens such as *Porphyromonas gingivalis*, *Treponema denticola*, and *Fusobacterium nucleatum* have been implicated as potential contributors to AD pathogenesis. Specifically, *P. gingivalis*, a primary periodontal pathogen, has been associated with accelerated AD pathologies, including amyloid-beta (Aβ) deposition and neuroinflammation [270]. Elevated antibody levels against periodontal bacteria such as *P. gingivalis*, *P. melaninogenica*, and Campylobacter rectus observed in patients with AD further indicate a relationship between bacterial exposure and AD risk [271,272,273,274,275]. Additional periodontal pathogens, including *Fusobacterium nucleatum*, *Prevotella intermedia*, *Actinomyces naeslundii*, and *Eubacterium nodatum*, have been suggested as potential biomarkers for the early diagnosis of AD, as antibodies to these pathogens were detected in individuals years before cognitive decline appeared [276,277,278].

The proposed mechanisms for the progression of AD include microbial invasion and systemic inflammation. Periodontal pathogens can access the brain through the bloodstream or neural pathways, initiating a series of events that exacerbate the pathology of AD. Their lipopolysaccharides (LPS) and gingipains activate inflammatory responses that contribute to neurodegeneration [279]. Animal studies corroborate these findings, where repeated exposure to *P. gingivalis* was linked to neurodegeneration and Aβ accumulation in the hippocampus, a hallmark of AD [279,280]. Furthermore, Dominy et al. identified that small-molecule inhibitors targeting gingipains may serve as a promising treatment to combat brain inflammation and bacterial colonization with *P. gingivalis*, which could slow the progression of neurodegeneration [279]. Moreover, in vitro research has demonstrated that periodontal bacteria LPS can reach the brain, potentially driving AD-associated inflammation [281].

Inflammatory mediators related to periodontal disease, including cytokines IL-1β, IL-6, and TNFα, have also been suggested to promote neuroinflammation, accelerating AD progression [282,283]. In particular, individuals with severe periodontitis show elevated plasma Aβ levels, possibly due to weakened vascular integrity that facilitates Aβ deposition in brain regions susceptible to AD [284]. Other hypotheses implicate the APOE-4 allele in increased susceptibility to AD, positing that APOE-4 may support the colonization of oral pathogens within the brain [285,286,287].

Alterations in oral microbiome composition, such as increased levels of Moraxella, Sphaerochaeta, and Leptotrichia, and a decline in beneficial bacteria like Rothia have also been observed as potential early indicators of AD [288,289,290]. Gram-negative bacteria from the oral cavity contribute to LPS production, potentially exacerbating Aβ plaque formation in AD [291,292]. Collectively, these findings highlight the significance of maintaining oral health as a key factor in the potential reduction in AD risk. Further longitudinal and interventional studies are needed to investigate these associations and determine whether the management of oral pathogens could mitigate neurodegenerative progression in AD [262,275,277,293].

## 19. Genes and AD

Over time, genetic factors have been established to play a significant role in the development of AD, accounting for 70% of cases. Therefore, Alzheimer’s disease can be categorized into familial and sporadic forms. The familial form, which accounts for 1–5% of cases, presents as early-onset (EOAD), occurring in patients under 65 years old, and is typically caused by mutations in PSEN1 (80% of cases), PSEN2 (5% of cases), and APP (15% of early-onset cases). In contrast, the sporadic form of AD, which accounts for 95% of cases, manifests itself as late-onset (LOAD), occurring in individuals over 65 years old, and is primarily associated with the APOE gene polymorphism, particularly the presence of the “4” allele. This allele is linked to an increased risk of developing Alzheimer’s disease three times in heterozygotes and twelve times in homozygotes [13,24,294]. Furthermore, it is known that PSEN1 mutations can lead to the most severe forms of AD, exhibiting complete penetration, with the onset of the disease occurring from 25 years of age [295]. Studies have shown that mutations in both the APP and PSEN1 genes result in the accumulation of amyloid-beta within the brain by elevating the Aβ42/40 ratio. Moreover, mutations in the APP gene may also elevate overall tau and phosphorylated tau concentrations within neurons [13,295,296].

The APOE gene, especially its “4” allele, which encodes the APOE protein, is the most significant genetic susceptibility factor for Alzheimer’s disease [297,298]. Apolipoprotein E (APOE) plays a vital role as a lipid carrier, facilitating communication between the blood–brain barrier (BBB) and the rest of the body. Any disturbance of this protein on either side of the BBB could potentially influence the development of Alzheimer’s disease [299]. Therefore, ApoE4 has been shown to play a role in the development of Alzheimer’s disease through various mechanisms. As a result, postmortem examinations of brain tissue from patients with Alzheimer’s disease have revealed that APOE4 exacerbates the accumulation of intra-neuronal Aβ, initiates the formation of Aβ oligomers, and diminishes Aβ clearance through microglial phagocytosis as well as the drainage of interstitial fluid (ISF) [9,223,300]. Moreover, APOE has been implicated in initiating cerebral amyloid angiopathy (CAA), impairing glucose metabolism and brain insulin signaling, influencing lipid transportation, interfering with mitochondrial function and synaptic plasticity, and promoting neuroinflammation and tauopathy in the brain [9,301]. Consequently, therapies specifically designed to target the ApoE ε4 allele have shown success in preventing Alzheimer’s disease [9].

It has been demonstrated that a significant number of these genes exhibit elevated levels of expression in the hippocampus, which is the primary neuroanatomical area impacted in Alzheimer’s disease. Moreover, certain genes could be involved in multiple mechanisms associated with AD, reinforcing the idea that both the disease itself and its treatment should be approached from a perspective that considers multiple converging factors, rather than focusing only on isolated mechanisms [13].

Therefore, these genes may be implicated in various neuropathological processes, including amyloid-beta production, aggregation, degradation, and clearance (involving genes such as ADAM10, CLU, CD33, PICALM, PTK2B, among others), tau pathology (BIN1, PICALM, CD2AP, MAPT, PTK2B, IGF1, and several other genes), the regulation of dendritic structure (CD2AP and COBL), axonal growth and transport (FERMT2 and CASS4), oxidative stress response (TOMM40, MEF2C, and MINK1), mitochondrial dysfunction (TOMM40), synaptic dysfunction (PICALM, PTK2B, and SLC10A2), lipid metabolism (PICALM, CLU, and SLC10A2), neuroinflammation (BIN1, CLU, TREM2, MTHFR, and MEF2C), microglial activation (MSHA6A, CD33, TREM2, and PLCG2), phagocytosis (ABCA7, INPPSD, and SCIMP), amyloid angiopathy (HLA-DRB5/HLA-DRB1), and blood–brain barrier disruption and vascular injury (CD2AP, EPHA1, and MTHFR). As a result, these genes may offer a significant opportunity for the development of early indicators and therapeutic targets in Alzheimer’s disease [13].

On the other hand, Alzheimer’s disease appears to exhibit a disturbance in epigenetic regulation. This is evidenced by diminished immunoreactivity and decreased 5-methylcytosine levels, which may indicate an inverse correlation with the presence of neurofibrillary tangles in cortical neurons observed in postmortem brain tissue samples from patients with Alzheimer’s disease [8].

Given the importance of genes in the pathogenesis of AD, modern therapies are increasingly focused on specialized techniques such as CRISPR-Cas9-mediated genome editing. These techniques have the capability to reverse mutations in various genes, including Bace1, APOEe4, APP, PSEN1, and PSEN2, using both viral and nonviral vectors. As a result, this appears to be a promising strategy for reducing amyloid-β deposits in the brain, not only in early-onset AD but also in the late-onset form of the disease [8,302,303].

In addition, current research on gene therapy for Alzheimer’s disease explores a range of innovative strategies that target key pathways implicated in neurodegeneration [304,305,306,307,308,309]. Therefore, in the United States, a phase 1 clinical trial (NCT05040217) is currently underway, investigating the administration of brain-derived neurotrophic factor (BDNF) into the brain using AAV2-BDNF vectors in patients with early AD or mild cognitive impairment (MCI). The purpose of this trial is to evaluate the safety, tolerability, and effectiveness of AAV2-BDNF gene therapy, in addition to its impact on disease progression biomarkers and cognitive results [306].

Moreover, apolipoprotein E2 (APOE-2)-targeted gene therapy, particularly for APOE ε4 carriers, has demonstrated promise in preclinical AD models. The delivery of APOE ε2 in these models has shown reductions in amyloid burden, neuroinflammation, and loss of synapses without microglial activation, offering protection against AD pathology [305]. Additionally, a phase 1/2 clinical trial on APOE-2 gene therapy (NCT03634007) is investigating the effectiveness and safety of LX1001 administration in patients homozygous to APOE4 through an intrathecal injection with an AAV gene transfer vector. The trial follows a dose-escalation design with three cohorts, and initial data from the low-dose cohort have shown positive outcomes, including APOE2 expression, biomarker reduction, and a well-tolerated profile [306]. Further studies in APOE-2 gene therapy are in development, including one with LX1021, which intended to express a modified Christchurch APOE-2 protein, which has protective properties, in the central nervous system (CNS) of APOE-4 homozygous patients. Another trial involves LX1021 with a dual approach: the expression of the protective APOE-2 protein in the central nervous system, alongside microRNA (miRNA) to reduce APOE-4 expression [306].

Research is also progressing on human telomerase reverse transcriptase (hTERT)-based gene therapy for AD. Therefore, a clinical trial (NCT04133454) aims to evaluate the safety and efficacy of LGT gene therapy (AAV-hTERT). In this study, participants receive a dose of LGT through intravenous and intrathecal administration, with a 12-month monitoring period for side effects, hTERT expression, and telomerase function, with the aim of clarifying the role of hTERT in neuroprotection and AD progression [306].

Furthermore, the neuroprotective potential of the nerve growth factor (NGF) has been examined in several gene therapy studies. A small phase 1 clinical trial involving eight subjects with early-stage AD observed a 36–51% reduction in disease progression over approximately two years, without side effects following the NGF gene to the cholinergic basal forebrain through stereotaxic administration [307]. Additional studies with AAV2-mediated NGF delivery have shown promising initial outcomes, including sustained, targeted NGF expression and biological activity, together with the cholinergic neuronal enlargement and growth of new axons up to a decade after transfer. However, larger trials, such as NCT00876863, have faced challenges, with inconclusive cognitive benefits potentially due to difficulties in vector targeting. Advances in real-time MRI guidance and convection-enhanced delivery are anticipated to improve targeting precision, facilitating robust evaluations of NGF’s therapeutic potential [308,309].

Other trials focus on the potential of gene therapies based on the neurotrophic factor (NF) in AD. A recent study investigates the hippocampal delivery of the NF-α1/CPE gene via AAV vectors in an AD mouse model, notably decreasing amyloid precursor protein (APP) expression, levels of insoluble Aβ1-42, and tau hyperphosphorylation, critical markers of AD. Treated mice exhibited better cognitive abilities, less neurodegeneration, and improved mitochondrial function and cell survival mechanisms, suggesting that NF-α1/CPE gene therapy could provide multiple benefits by strengthening cell survival pathways and reducing inflammation [304].

In conclusion, these gene therapy strategies offer promising molecularly targeted approaches for AD treatment. Continued research is necessary to refine the delivery techniques, enhance therapeutic efficacy, and thoroughly assess the long-term safety and clinical potential of these therapies [304,305,306,307,308,309]. 

Genes may also provide a significant opportunity to develop individualized therapy for patients, considering specific genes associated with distinct stages of the disease and particular regions of the brain [7].

## 20. The Overlap Between AD and Parkinson’s Disease (PD)

**Figure 3:**
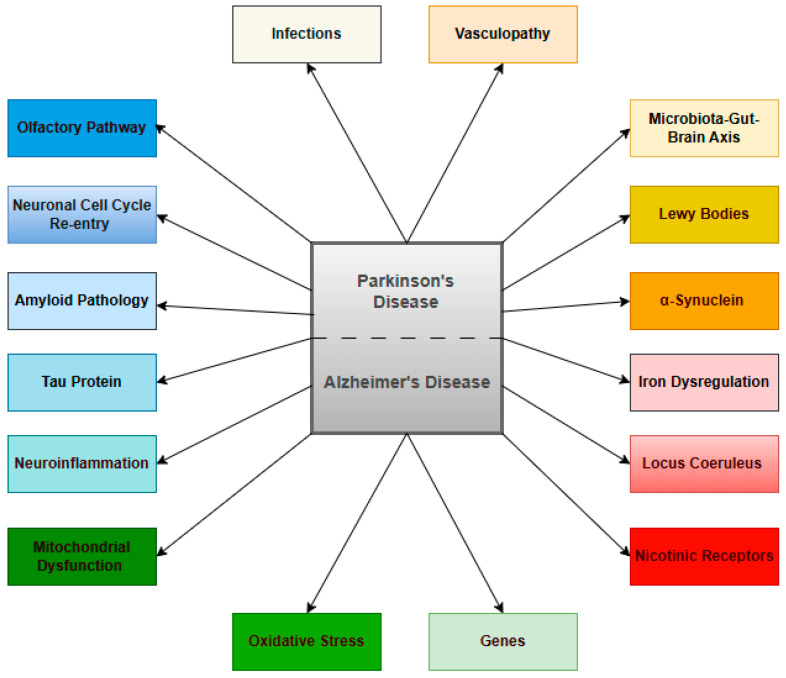
The overlap mechanisms between AD and PD.

Parkinson’s disease (PD), the second most prevalent neurodegenerative disorder after AD, shares multiple pathogenic mechanisms with AD, underscoring a complex etiopathogenetic overlap (see Figure 3). Both diseases display pathology involving amyloid deposits, tau protein aggregation, neuroinflammation, mitochondrial dysfunction, oxidative stress, nicotinic receptors, locus coeruleus degeneration, α-synuclein accumulation with Lewy bodies, iron dysregulation, microbiota–gut–brain axis disruptions, infectious agents, vascular pathology, neuronal cell cycle re-entry, olfactory deficits, and genetic susceptibility [310,311,312,313,314,315,316,317].

Although AD is typically defined by amyloid-beta (Aβ) plaque accumulation, but similar deposits are found in PD, particularly in advanced stages with dementia. Cross-seeding interactions between Aβ and α-synuclein are shown to exacerbate the progression of PD, suggesting that Aβ pathology influences PD and may drive neurodegenerative processes common to both diseases [318].

Tau protein, known to form neurofibrillary tangles in AD, is also implicated in PD. Studies reveal a correlation between tau and α-synuclein levels in early-stage PD, and tau-α-synuclein co-aggregation likely facilitates the propagation of abnormal protein assemblies, contributing to pathology in both diseases [319].

Chronic inflammation and oxidative stress are prominent in both AD and PD, where activated microglia and astrocytes promote neurodegeneration through cellular damage and death. This inflammatory response appears to contribute to overlapped immune pathway dysfunctions in both disorders [316].

Moreover, mitochondrial dysfunction, a critical factor in the pathogenesis of AD and PD, involves mutations in mitochondrial DNA that increase reactive oxygen species (ROS) production, exacerbating oxidative stress and promoting neurodegeneration through energy deficits and ROS-induced cell injury [316].

In addition, nicotinic acetylcholine receptors (nAChRs), which regulate neurotransmission in the central nervous system, are involved in both AD and PD. The pharmacological modulation of nAChR activity is under investigation for therapeutic intervention in these diseases [320]. The significant loss of neurons in the locus coeruleus (LC), a primary source of brain norepinephrine, occurs in both PD and AD. Early LC degeneration can contribute to symptoms such as cognitive impairment, as norepinephrine deficiency affects the arousal and cognitive processes shared by both disorders [321,322,323]. α-Synuclein misfolding and aggregation, pivotal in PD pathology, also affect AD by potentially disrupting synaptic function and aggregating in Aβ-rich regions. The coexistence of AD and PD pathology in Lewy body dementia (LBD) exemplifies the cross-disease impact of α-synuclein [324,325].

Furthermore, iron accumulation is observed in regions severely affected by AD and PD. By catalyzing oxidative stress, iron enhances α-synuclein aggregation in PD and amyloid plaque formation in AD, contributing to neurotoxicity in both conditions [326].

The gut–brain axis is also involved in PD pathogenesis, as gastrointestinal symptoms often precede motor symptoms, suggesting that this axis could influence neuroinflammation and disease progression in both PD and AD [312,313,314]. Additionally, chronic inflammation and infection can further promote neurodegeneration in both diseases. Such inflammatory states can serve as potential triggers that accelerate pathology through bidirectional propagation mechanisms [317].

Vascular changes, including white matter hyperintensities (WMHs), are linked to cognitive and motor decline in AD and PD, as cerebrovascular insufficiency can impede amyloid clearance and contribute to cognitive impairment and general neurodegeneration in both disorders [310]. Recently, neuronal cell cycle re-entry (CCR) is gaining recognition as a feature of neurodegeneration, with evidence that in PD, neurons aberrantly attempt cell division, leading to neuronal death [311].

Olfactory dysfunction is another early symptom shared by AD and PD. In PD, olfactory impairment may appear years before motor symptoms, suggesting the olfactory pathway as an early neurodegeneration site in both conditions [315].

Although genetic overlap between AD and PD is limited, heritability studies indicate shared microglial-related genetic regions. Common genetic variants in genes such as GSTO1, GSTO2, and NEDD9, which are involved in the regulation of oxidative stress, demonstrate a minor genetic correlation, particularly at immune-related loci, linking genetic susceptibility to both neurodegenerative diseases [327].

In conclusion, the complexity of the overlapping mechanisms in AD and PD suggests that these diseases may not only share common pathological pathways but also could benefit from therapeutic strategies addressing these shared processes.

## 21. Conclusions

Although multiple mechanisms have been described in the pathogenesis of AD, the precise etiopathogenesis of this disorder remains unclear. There appears to be an overlap in the risk factors associated with this condition, as many of these mechanisms are interconnected, ultimately culminating in neuronal degeneration.

As a result, newly described mechanisms in Alzheimer’s pathogenesis, such as the serotoninergic system, autophagy, vascular dysfunction, the metal hypothesis, microbiota, glymphatic, lymphatic systems, the olfactory pathway, and the oral health, provide a significant opportunity to comprehend the underlying pathology of Alzheimer’s disease and explore new potential therapies. On the other hand, most of the classical mechanisms, which have also been widely investigated, may offer new therapeutic targets by promoting various molecules implicated in different neuropathological processes.

Despite the numerous compounds currently in various stages of research, there are very few FDA-approved disease-modifying drugs for AD, with only two new monoclonal antibodies in clinical use since 2023. Although monoclonal antibodies are highly effective in reducing the Aβ accumulation in the brain, their practical clinical effect is limited, as they do not cure Alzheimer’s disease but merely slow its progression. Additionally, mAbs are associated with major side effects, including considerable risks of brain swelling and bleeding, impacting their suitability for broad clinical application [328,329,330].

Recently, there has been increasing interest in probiotics and antioxidant compounds as a novel approach to this disorder. Additionally, nanoparticle-based therapy, neural stem cell transplantation, vaccines, and CRISPR-Cas9-mediated genome editing techniques have also emerged as areas of interest.

In conclusion, future research should primarily focus on developing combination therapies that are capable of targeting multiple mechanisms of the disease simultaneously. Although several molecules, such as edaravone, are being studied, none of them are currently approved for clinical use. A future perspective on Alzheimer’s disease could also involve individualized therapy that targets specific phases of the disease and particular brain regions. This novel and promising strategy may be facilitated by genes.

## Figures and Tables

**Table 2:** Microglial therapeutic targets in Alzheimer’s disease.

Therapeutic Target	Underlying Molecular Mechanism	Mode of Molecular Interference	References
Notch signaling pathway	Dysregulation of microglial activation, with a bias toward a pro-inflammatory phenotype, influencing the production and the clearance of amyloid-beta (Aβ) plaques	Restoring balance in microglial states and alleviating neuroinflammation	[78]
CX3CL1/CX3CR1 pathway	Microglia-mediated tau pathology (low levels of CX3CL1 and CX3CR1 in AD)	Modulation of CX3CL1 and CX3CR1 levels within the hippocampus and frontal cortex	[80]
NLRP3 inflammasome	Neuroinflammation and neuronal damage	Decreasing Tau phosphorylation and Aβ accumulation in the hippocampus of TauP301S transgenic mice through inhibition of NLRP3	[77,85]
PPAR-γ	Modulation of pro-inflammatory and anti-inflammatory cytokines and regulation of autophagy	- PPAR-γ agonists (pioglitazone): decreasing the production of pro-inflammatory cytokines - PPAR-γ antagonists: promoting a shift in microglial activation toward a regulatory and reparative phenotype by increasing autophagy through the LKB1/AMPK signaling pathway	[78,84]
RIPK1	TNF-α-induced necroptosis pathway	Facilitating the degradation of amyloid-beta (Aβ) by microglia through inhibition of RIPK1	[86]
CALHM2	Regulation of calcium influx	Reducing neuroinflammation and accumulation of Aβ through CALHM2 inhibition	[87]
CD33	Impaired phagocytic function of microglia induced by up-regulation of CD33 expression	Reversing altered microglial phagocytic function concerning Aβ through inhibition of CD33	[78,83,84]
TREM2	Facilitating the phagocytic activity of microglia specific to Aβ	- Overexpression of human TREM2: reducing amyloid plaque deposition - Agonist antibodies targeting TREM2: reducing Aβ burden	[77,78,84]
MAPK, TLR, JAK/STAT, NF-κB, PI3K/AKT signaling pathways	Microglial activation pathways	Modulation of neuroinflammation through interference with microglial activation	[78]
